# Simplified theory to predict contact ion pair formation in electrolyte mixtures containing hydroxylammonium nitrate

**DOI:** 10.1371/journal.pone.0358491

**Published:** 2026-09-23

**Authors:** Benjamin Shaw

**Affiliations:** Mechanical and Aerospace Engineering Department, University of California, Davis, California, United States of America; Monash University, AUSTRALIA

## Abstract

Simplified theory is applied to predict ${\text{NH}}_{3}{\text{OH}}^{+}\cdots {{\text{NO}}_{3}}^{-}$ contact ion pair (CIP) concentrations in electrolyte mixtures containing hydroxylammonium nitrate (HAN). The temperature range considered is 300 K−523 K. The theory considers HAN loadings in the condensed phase less than 1 *M*, which is where some experiments to investigate aqueous HAN decomposition kinetics have been performed. It is found that when HAN is mixed with methanol or ethanol, CIP concentrations can peak at small nonzero levels of the initial HAN loading, while HAN-water mixtures are predicted to not exhibit such CIP concentration peaks. These results are relevant to increasing understanding of conditions under which chemical reactivity as well as thermodynamic and transport properties may be influenced by the presence of CIPs.

## Introduction

Hydroxylammonium nitrate (HAN, chemical formula NH_3_OHNO_3_) based mixtures are important in plutonium processing systems [1]. HAN is also a major constituent in a class of liquid monopropellants that have many attractive characteristics and which display phenomena that differ significantly from other liquid monopropellants [2]. HAN-based propellants have attracted attention as liquid gun propellants [2] and for spacecraft propulsion applications [3]. HAN-based propellants are composed primarily of HAN, H_2_O, and a fuel species. For example, methanol, ethanol, glycine, and triethanolammonium nitrate (TEAN) have been investigated as potential fuel species in HAN-based monopropellants [4–12].

As a salt, HAN can dissociate into ions when in solution. A general characteristic of electrolyte solutions is ion association, where oppositely charged ions are attracted to each other to form associated ions [13–16]. For example, it is possible to form ion pairs, as denoted in Eq. (1), where the symbol ⇌ denotes equilibrium and NH_3_OH^+^⋯ NO_3_^–^ denotes the associated ion pair.


$$\begin{array}{c} {\text{NH}}_{3}{\text{OH}}^{+}+{{\text{NO}}_{3}}^{-}⇌{\text{NH}}_{3}{\text{OH}}^{+}\cdots {{\text{NO}}_{3}}^{-} \end{array}$$ (1)

The associated ions are attracted together from differences in charge. The presence of associated ions can influence electrolyte properties such as electrical conductivity, viscosity, and chemical reactivity. In this analysis we consider contact ion pairs (CIPs), i.e., where the ions in an ion pair are in direct contact.

An important initiation reaction for HAN decomposition in solution is proton transfer between the NH_3_OH^+^ and NO_3_^–^ ions [17,18], as shown in Eq. (2).


$$\begin{array}{c} {\text{NH}}_{3}{\text{OH}}^{+}+{{\text{NO}}_{3}}^{-}⟶{\text{NH}}_{3}O+{\text{HNO}}_{3} \end{array}$$ (2)

The products of this reaction can react further, releasing the heat associated with decomposition. When contact ions are present in solution, the NH_3_OH^+^ and NO_3_^–^ ions are in close proximity, which may increase the likelihood of the occurrence of Eq. (2). This is because the rate of proton transfer from NH_3_OH^+^ to NO_3_^–^ will depend on intervening species. When the ions are completely solvated, a proton has to move through the solvation layer(s), which may require a higher activation energy than for the case where the ions are in direct physical contact. As a result, factors that increase the CIP concentration may also influence the reactivity of HAN in solution.

Previous research on HAN decomposition chemistry has concluded that CIP concentrations are negligible for aqueous solutions with low HAN loadings, and chemical kinetic mechanisms have been developed based on this conclusion [19,20]. The primary hypothesis of this research is that solvents with low dielectric constants can increase ion association sufficiently for contact ion pairs to potentially become relevant to HAN decomposition chemistry, even at dilute HAN loadings. The present research demonstrates that neglecting CIP formation may not be appropriate for liquids that have low dielectric constants relative to water, e.g., methanol or ethanol, which are considered here. In addition, the dilute regime investigated here isolates the thermodynamic factors controlling contact ion pair formation and provides useful initial conditions for fundamental kinetic studies. The practical relevance of this research is that low-dielectric solvents may create chemically distinct initial speciation states, and chemical kinetic models developed under these conditions may need to account for CIPs.

This research is focused on dilute HAN loadings, i.e., less than 1 *M*. It is expected that HAN is completely soluble in ethanol, methanol, and water under the conditions investigated here. For example, Amrousse et al. [12] report experimental results on studies of HAN mixed with ethanol, methanol, and water with HAN loadings greater than 1 *M*.

## Theory for ion pair concentrations

An ion pair is often classified as being a solvent-separated ion pair (SSIP), a solvent-shared ion pair (SIP), or a contact ion pair (CIP). An SSIP has two solvent molecules between the ions, an SIP has one solvent molecule between the ions, and with a CIP the ions are in direct contact. If the hard-sphere diameter for a solvent molecule is $\sigma_{s}$, then the ions in an SSIP are separated by the distance $2\sigma_{s}$ while with an SIP they are separated by the distance $\sigma_{s}$. Table 1 shows hard-sphere diameter values that were found in the literature.

**Table 1:** Effective hard-sphere diameters used for solvent molecules and ions.

Species	σ(nm)	Reference
$H_{2}O$	0.280	[21]
$CH_{3}OH$	0.383	[21,22]
$C_{2}H_{5}OH$	0.444	[21,22]
$NO_{3}^{-}$	0.340	[23]
$NH_{3}OH^{+}$	0.350	[24,25]

In the present context, we identify the chemical formula for the SSIP as $A^{+}S_{2}B^{-}$, the SIP as $A^{+}SB^{-}$, and the CIP as $A^{+}B^{-}$, where *S* denotes a solvent molecule. With this model, the formation of a CIP is assumed to occur sequentially via the following equilibrium reactions


$$\begin{array}{c} {\text{A}}^{+}+{\text{B}}^{-}+2\text{S}\overset{{\text{K}}_{1}}{⇌}\text{SSIP} \end{array}$$ (3)

$$\begin{array}{c} SSIP\overset{{\text{K}}_{2}}{⇌}SIP+S \end{array}$$ (4)

$$\begin{array}{c} SIP\overset{{\text{K}}_{3}}{⇌}CIP+S \end{array}$$ (5)

where *K*_1_, *K*_2_, and *K*_3_ are equilibrium constants. These may be expressed as follows


$$\begin{array}{c} K_{1}=\frac{\left[SSIP\right]}{[A^{+}][B^{-}][S{]}^{2}}\frac{1}{f_{\pm }^{2}} \end{array}$$ (6)

$$\begin{array}{c} K_{2}=\frac{\left[SIP][S\right]}{\left[SSIP\right]} \end{array}$$ (7)

$$\begin{array}{c} K_{3}=\frac{\left[CIP][S\right]}{\left[SIP\right]} \end{array}$$ (8)

where the activity coefficients of the SSIP, the SIP, the CIP, and the solvent are all assumed to be unity. Rearranging these expressions yields


$$[SSIP]=K_{1}[A^{+}{]}^{2}f_{\pm }^{2}$$ (9)

$$[SIP]=K_{1}K_{2}[A^{+}{]}^{2}f_{\pm }^{2}$$ (10)

$$[CIP]=K_{1}K_{2}K_{3}[A^{+}{]}^{2}f_{\pm }^{2}$$ (11)

where it has been stipulated that $\left[A^{+}]=[B^{-}\right]$. An atom balance yields


$$[A^{+}]+[SSIP]+[SIP]+[CIP]=[AB{]}_{0}.$$ (12)

Inserting Eqs. 9, 10, and 11 into Eq. 12 yields the following expression for [*A*^+^].


$$\begin{array}{c} f_{\pm }^{2}K[A^{+}{]}^{2}+[A^{+}]=[AB{]}_{0} \end{array}$$ (13)

where


$$\begin{array}{c} K=K_{1}+K_{1}K_{2}+K_{1}K_{2}K_{3}. \end{array}$$ (14)

The *K*_1_, *K*_2_, and *K*_3_ values may be evaluated using the Bjerrum, Ebeling, or Fuoss theories. Specifically, $K_{1}=K_{SSIP}$, $K_{2}=K_{SIP}/K_{SSIP}$, and $K_{3}=K_{CIP}/K_{SIP}$ where the subscripts *SSIP*, *SIP*, and *CIP* indicate how the center-to-center ion distance is to be evaluated.

## Evaluation of the relative permittivity

Relative permittivities of the solvents were evaluated using data from [26]. Specifically [26], presents the empirical correlation


$$\begin{array}{c} \epsilon =A_{0}+A_{1}T+A_{2}T^{2}+\frac{A_{4}}{T}+A_{5}\ln T \end{array}$$ (15)

where *A*_0_, *A*_1_, *A*_2_, *A*_4_, and *A*_5_ are constants and *T* is absolute temperature. The constants for water, methanol, and ethanol, as reported by [26], are in Table 2.

**Table 2:** Relative permittivity correlation coefficients [26].

Substance	*A*_0_	*A*_1_	*A*_2_	*A*_4_	*A*_5_
Water	−1664.4988	−0.884533	0.0003635	64839.1736	308.3394
Methanol	−1750.3069	−0.990260	0.0004666	51360.2652	327.3124
Ethanol	−1522.2782	−1.005080	0.0005211	38733.9481	293.1133

**Fig 1:**
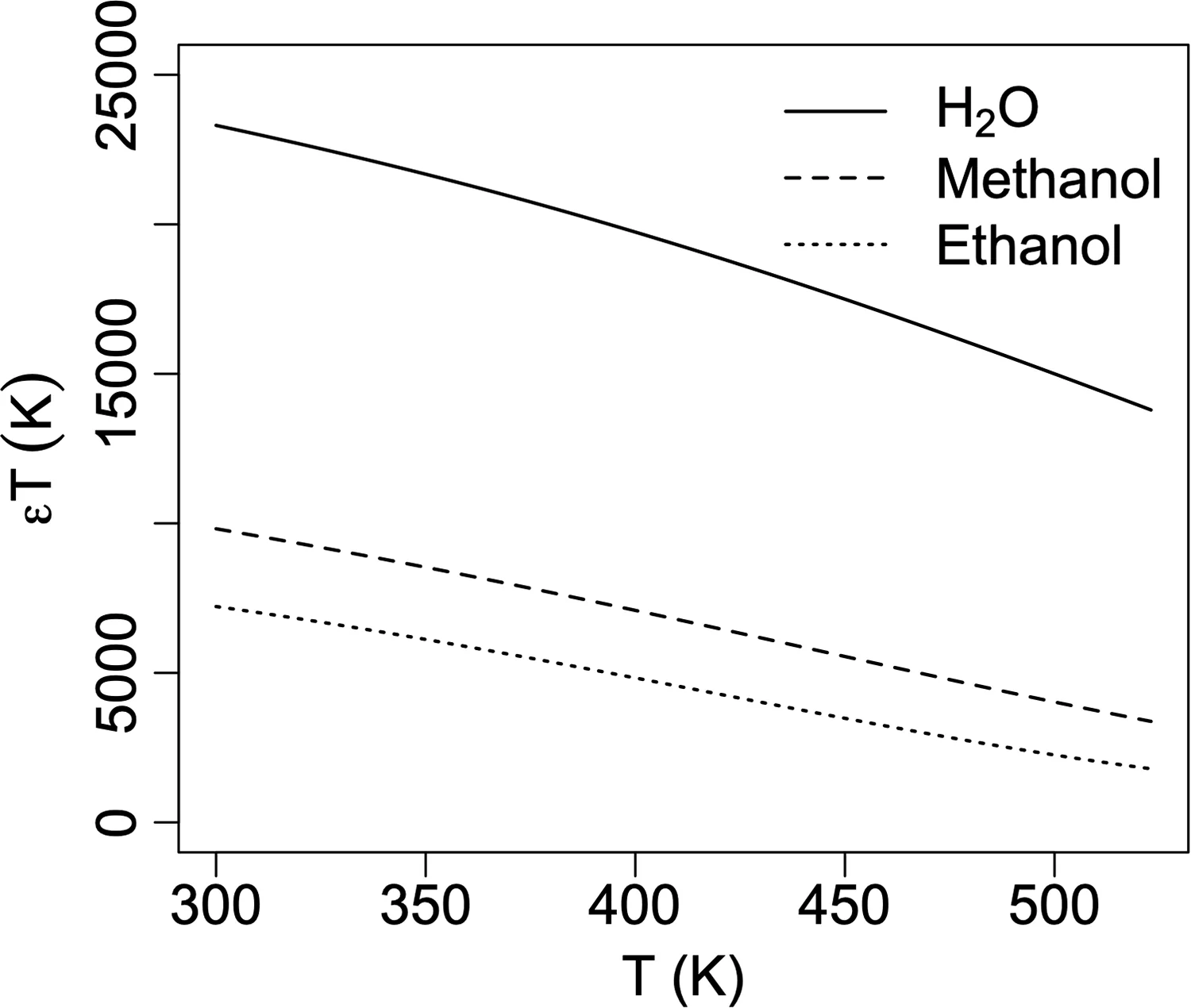
Plots of εT as a function of *T* for water, methanol, and ethanol.

As will be shown later, ϵ and *T* always appear as a product in the relevant equations, so we provide data on how this product varies with temperature. Fig 1 (S1 Fig and S1 File) shows plots of εT as a function of *T* for liquid water, liquid methanol, and liquid ethanol over the temperature range 300 *K* – 523 *K*, based on the values in Table 2. The εT values for water range from about 14000 *K* to 23000 *K*. For comparison, the εT values for methanol and ethanol are significantly smaller, varying over the approximate ranges 5000 *K* – 10000 *K* and 1800 *K* – 7000 *K*, respectively. In all cases, εT decreases as *T* increases.

Because we are concerned with low molarities, the influence of the electrolyte on the relative permittivity of the solution is assumed to be negligible.

Molecular-level simulations and spectroscopic measurements provide useful context for the ion-association processes considered here. Depew et al. [27] used molecular-dynamics simulations to investigate the liquid structure and hydrogen-bonding environment of aqueous HAN, while Zhou et al. [28] combined molecular-dynamics simulations with tandem mass spectrometry to examine HAN structure, proton transfer, and dissociation. Studies of related nitrate-containing systems also demonstrate that the solvent environment can strongly influence ion association. D’Angelo et al. [29] combined molecular-dynamics simulations with X-ray absorption spectroscopy and observed nitrate contact-ion-pair formation in methanol, and Kelley et al. [30] found that ion pairing becomes increasingly favorable as methanol content increases in water-methanol mixtures. Xu et al. [31] used molecular-dynamics calculations and Raman spectroscopy to distinguish contact, solvent-shared, and solvent-separated nitrate ion pairs in aqueous solutions. These studies support the physical plausibility of solvent-dependent ion association, but they do not provide direct validation of the predicted contact-ion-pair concentrations in dilute HAN-methanol or HAN-ethanol mixtures considered here. The absence of such direct molecular-level simulation or spectroscopic validation is a limitation of the present analysis.

## Evaluation of activity coefficients

There have been many expressions proposed for evaluation of the activity coefficients of an ion, e.g., the Debye-Huckel (DH), Extended Debye-Huckel (EDH), Truesdell-Jones (TJ), and associative mean spherical approximation (AMSA) equations. Because a contact ion pair is electrically neutral, it is assumed that *f*_0_ = 1.

The DH equation for the activity coefficient of species *i* is given by


$$\begin{array}{c} \ln f_{i}=-\alpha z_{i}^{2}I^{1/2} \end{array}$$ (16)

where *I* is the ionic strength of the solution, $z_{i}$ the charge of an individual ion *i*, and α is as follows.


$$\begin{array}{c} \alpha =\frac{(2N_{0}{)}^{1/2}e_{0}^{3}}{8\pi (\epsilon_{0}k_{b}{)}^{3/2}}\frac{1}{(\epsilon T{)}^{3/2}} \end{array}$$ (17)

The ionic strength is defined as


$$\begin{array}{c} I=\frac{1}{2}\sum_{i}z_{i}^{2}[i] \end{array}$$ (18)

where [*i*] is the molar concentration of species *i*. The DH equation is reported to be accurate for *I* < 0.005 *M*.

As noted by Fraggedakis [32], it is useful to express the activity coefficient models in terms of the Bjerrum and Debye lengths. The Bjerrum length, $\lambda_{B}$, is the distance away from an ion where the characteristic random thermal energy $k_{b}T$ equals the characteristic electrostatic energy $e_{0}^{2}/4\pi \epsilon_{0}\epsilon \lambda_{B}$.


$$\begin{array}{c} \lambda_{B}=\frac{e_{0}^{2}}{4\pi \epsilon_{0}k_{b}\epsilon T} \end{array}$$ (19)

The Debye length, $\lambda_{D}$, is a characteristic distance over which the electric field of an ion persists before being screened by surrounding charges.


$$\begin{array}{c} \lambda_{D}={\left(\frac{\epsilon_{0}k_{b}\epsilon T}{2N_{0}e_{0}^{2}I}\right)}^{1/2} \end{array}$$ (20)

The DH equation may be expressed in terms of $\lambda_{B}$ and $\lambda_{D}$ as follows.


$$\begin{array}{c} \ln f_{i}=-\frac{z_{i}^{2}}{2}\frac{\lambda_{B}}{\lambda_{D}} \end{array}$$ (21)

The EDH equation [33], i.e., Eq. 22 is as follows


$$\begin{array}{c} \ln f_{i}=\frac{-\alpha z_{i}^{2}I^{1/2}}{1+\beta a_{i}I^{1/2}} \end{array}$$ (22)

where $a_{i}$ is an empirical parameter intended to allow an ion to have a finite size instead of being a point charge, and


$$\begin{array}{c} \beta ={\left(\frac{2e_{0}^{2}N_{0}}{\epsilon_{0}k_{b}\epsilon T}\right)}^{1/2}. \end{array}$$ (23)

The EDH equation is reported to be accurate for *I* < 0.1 *M*. The EDH equation may be expressed in terms of $\lambda_{B}$ and $\lambda_{D}$ as follows.


$$\begin{array}{c} \ln f_{i}=-\frac{z_{i}^{2}}{2}\frac{\lambda_{B}}{\lambda_{D}}\frac{1}{1+a_{i}/\lambda_{D}} \end{array}$$ (24)

The TJ equation [33] is


$$\begin{array}{c} \ln f_{i}=\frac{-\alpha z_{i}^{2}I^{1/2}}{1+\beta a_{i}^{0}I^{1/2}}+\gamma I \end{array}$$ (25)

where γ is an empirical factor that is used to obtain better agreement with experiment for larger *I* values. The variable $a_{i}^{0}$ is an empirical factor that allows an ion to have a finite size. The TJ equation is reported to be accurate for *I* < 1 *M*. The TJ equation may be expressed in terms of $\lambda_{B}$ and $\lambda_{D}$ as follows.


$$\begin{array}{c} \ln f_{i}=-\frac{z_{i}^{2}}{2}\frac{\lambda_{B}}{\lambda_{D}}\frac{1}{1+a_{i}^{0}/\lambda_{D}}+\frac{\gamma }{8\pi N_{0}\lambda_{B}\lambda_{D}^{2}} \end{array}$$ (26)

The AMSA theory yields the following expression


$$\begin{array}{c} \ln f_{i}=\frac{-z_{i}^{2}e_{0}^{2}}{4\pi \epsilon_{0}k_{b}\epsilon T}\frac{\Gamma }{1+\Gamma \sigma_{i}} \end{array}$$ (27)

where Γ is found by solving


$$\begin{array}{c} 4\Gamma^{2}=\frac{e_{o}^{2}N_{0}}{\epsilon_{0}k_{b}\epsilon T}\sum_{i}\frac{z_{i}^{2}[i]}{(1+\Gamma \sigma_{i}{)}^{2}}. \end{array}$$ (28)

If all ions are assumed to have the same diameter σ, then Eq. 28 reduces to the following form


$$\begin{array}{c} \Gamma =\frac{(1+2\sigma \kappa {)}^{1/2}-1}{2\sigma } \end{array}$$ (29)

where


$$\begin{array}{c} \kappa ={\left(\frac{e_{0}^{2}N_{0}}{\epsilon_{0}k_{b}\epsilon T}\sum_{i}z_{i}^{2}[i]\right)}^{1/2}={\left(\frac{2e_{0}^{2}N_{0}}{\epsilon_{0}k_{b}\epsilon T}I\right)}^{1/2} \end{array}$$ (30)

and


$$\begin{array}{c} \ln f_{i}=\frac{-z_{i}^{2}e_{0}^{2}}{4\pi \epsilon_{0}k_{b}\epsilon T}\frac{\Gamma }{1+\Gamma \sigma }. \end{array}$$ (31)

The AMSA equation is reported to be accurate for I<2−3 M.

The AMSA equations become as follows in terms of $\lambda_{B}$ and $\lambda_{D}$ for distinct $\sigma_{i}$


$$\begin{array}{c} \ln f_{i}=-z_{i}^{2}\lambda_{B}\frac{\Gamma }{1+\Gamma \sigma_{i}} \end{array}$$ (32)

$$\begin{array}{c} \Gamma^{2}=\pi N_{0}\lambda_{B}\sum_{i}\frac{z_{i}^{2}[i]}{(1+\Gamma \sigma_{i}{)}^{2}} \end{array}$$ (33)

while for $\sigma_{i}=\sigma$ it follows that


$$\begin{array}{c} \kappa =\frac{1}{\lambda_{D}} \end{array}$$ (34)

$$\begin{array}{c} \Gamma =\frac{(1+2\sigma /\lambda_{D}{)}^{1/2}-1}{2\sigma } \end{array}$$ (35)

$$\begin{array}{c} \ln f_{i}=-z_{i}^{2}\frac{\lambda_{B}}{\sigma }\frac{(1+2\sigma /\lambda_{D}{)}^{1/2}-1}{(1+2\sigma /\lambda_{D}{)}^{1/2}+1}. \end{array}$$ (36)

**Fig 2:**
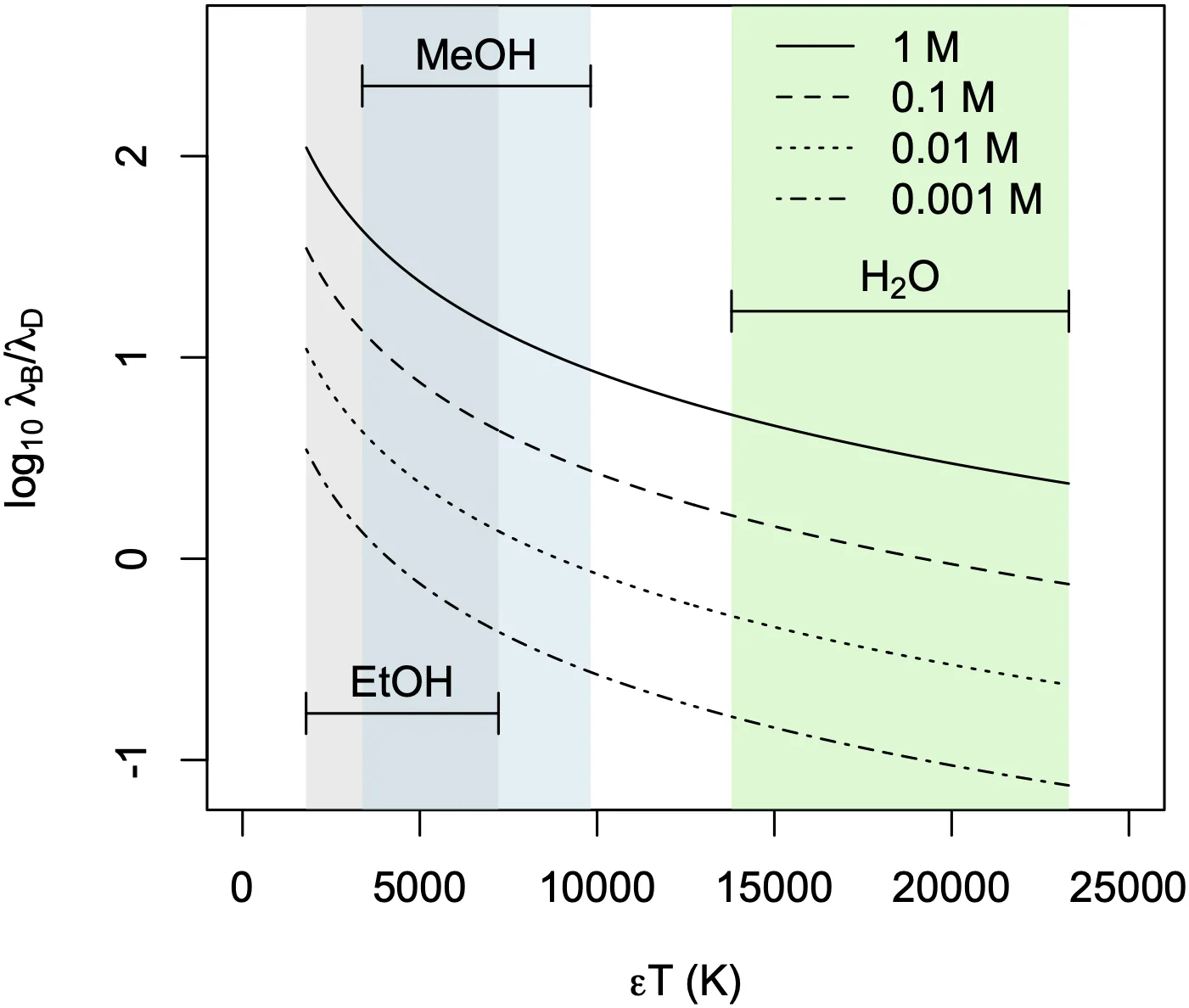
Plots of the ratio $\lambda_{B}/\lambda_{D}$ as a function of ϵT for different values of the ionic strength *I.*

Fig 2 (S2 Fig and S1 File) shows plots of the ratio $\lambda_{B}/\lambda_{D}$ as a function of ϵT and for different values of *I*. This figure shows that $\lambda_{B}/\lambda_{D}$ increases as ϵT decreases or *I* increases, indicating that CIPs should be more prevalent for ethanol and methanol relative to water for a given ionic strength.

**Fig 3:**
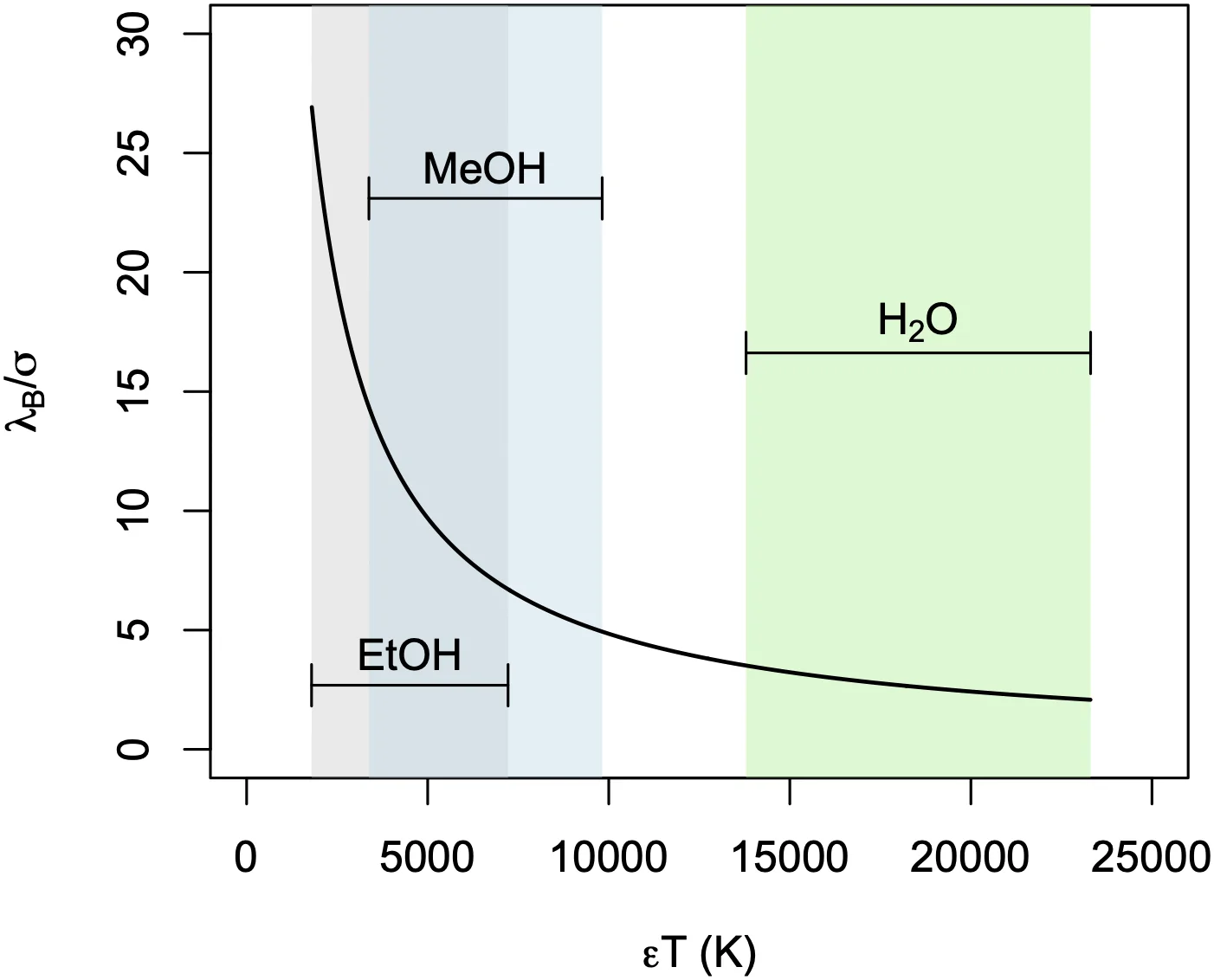
Plot of the ratio $\lambda_{B}/\sigma$ for σ=0.345 nm and different values of ϵT.

Another important quantity is the ratio of the Bjerrum length to the hard-sphere diameter of an ion, σ. Fig 3 (S3 Fig and S1 File) shows a plot of $\lambda_{B}/\sigma$ as a function of ϵT for σ=0.345 nm. For water, $\lambda_{B}/\sigma$ varies over the approximate range 2–4, but for methanol and ethanol, the ranges for $\lambda_{B}/\sigma$ are about 5–14 and 7–27, respectively.

The larger values of $\lambda_{B}/\lambda_{D}$ and $\lambda_{B}/\sigma$ for methanol and ethanol suggest that the AMSA approach is most appropriate for calculating the activity coefficient of an ion and that the other approaches may be less accurate. All of the approaches will be considered here, however, so that their predictions may be compared.

The theory we are using assumes that the dielectric constant is the same everywhere within the solvent. It is noted that the local dielectric environment near an ion is influenced by the presence of the ion itself. The high electric field strength near an ion will reduce the local dielectric constant in fluids such as water, methanol, and ethanol because the field will hinder movement of solvent molecules near the ion, reducing their ability to respond to an applied electric field, which is termed dielectric saturation. This phenomenon has been studied using continuum and field-theoretic approaches [34–37]. The electrostatic self-energy in inhomogeneous dielectric media has also been investigated [38]. If ions experience hydrogen bonding with solvent molecules, the solvent molecules would be hindered from responding to an applied electric field, which would also reduce the local dielectric constant.

In order to allow use of the simplified theory employed here, quantities such as the hard-sphere ion diameters may be considered to be parameters that can be adjusted to provide fits to experimental data. Activity coefficient data were not found for the $NH_{3}OH^{+}$ - *NO*3^–^ system, so we investigate a range of values for the hard-sphere diameters of these ions. For simplicity, the σ values for $NH_{3}OH^{+}$ and *NO*3^–^ are assumed to be equal.

**Fig 4:**
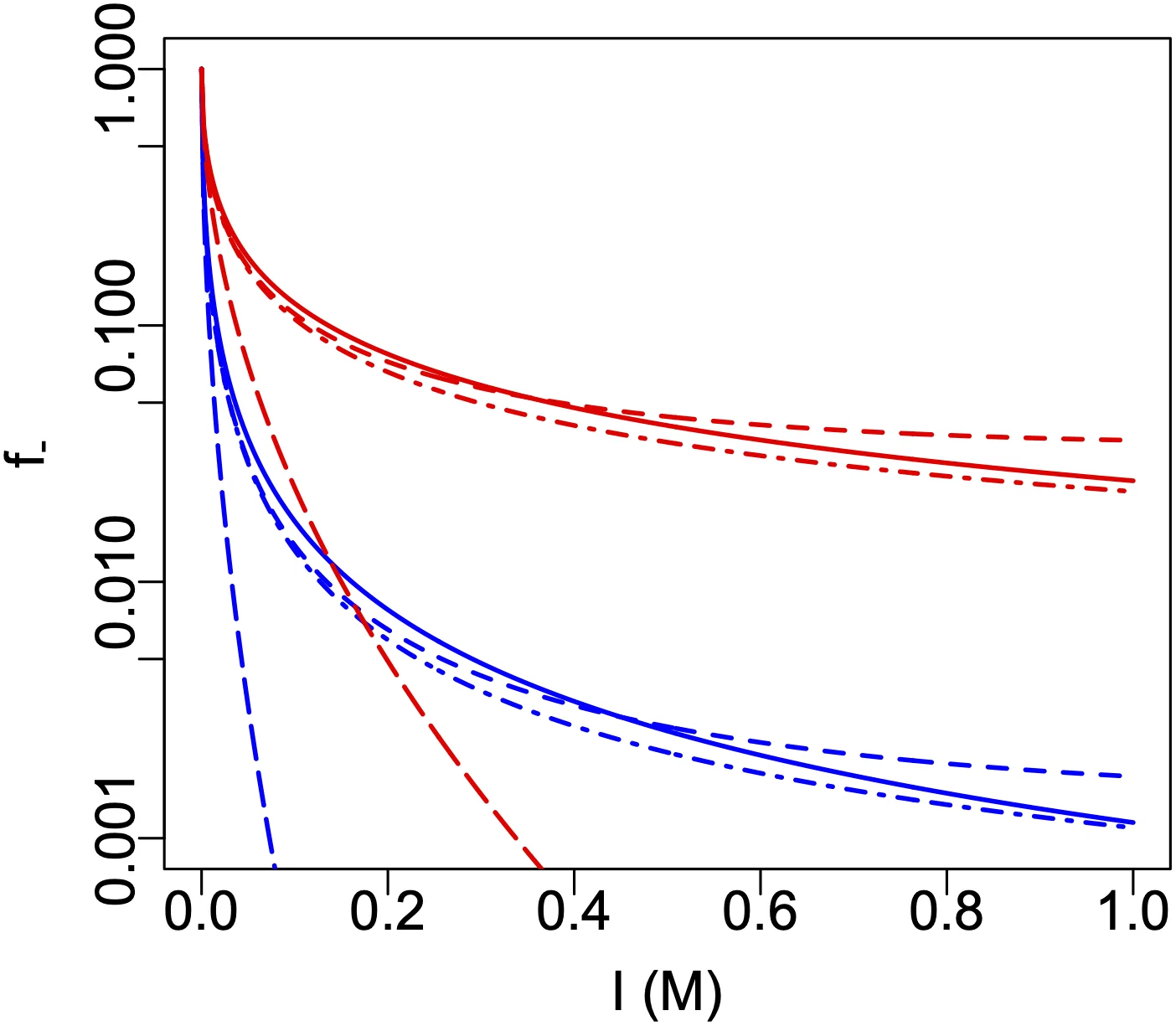
Plots of *f*_–_ for the AMSA equation (solid lines), the Truesdell-Jones equation (short dashed lines), the extended Debye-Huckel equation (dot-dash lines), and the Debye-Huckel equation (long dash lines) as a function of *I* for different εT values: blue curves εT=3000 K; red curves εT=5000 K.

Fig 4 (S4 Fig and S1 File) shows plots of *f*_–_ as a function of *I* for different values of ϵT for the Debye-Huckel equation, the extended Debye-Huckel equation, the Truesdell-Jones equation, and the AMSA equation. The activity coefficient changes rapidly for *I* near zero. The plots employed the values $a_{i}=a_{i}^{0}=0.3\;nm$, σ=0.4 nm and $\gamma =0.4605\;M^{-1}$ [33]. All of the models approach each other as *I* decreases. It is also noticeable that as ϵT decreases, the plots of *f*_–_ become very steep for small *I*.

## Evaluation of association constants

The association constant *K* is evaluated using the theories of Bjerrum [39], Ebeling [40], and Fuoss [41]. A review of these theories is available [42]. Related studies have considered concentrated electrolytes through ion-pair distribution functions and Poisson-Boltzmann-based descriptions of Bjerrum pairs and their effect on dielectric response and screening length [43–45]. Because we are focused here on dilute electrolyte solutions, we employ the simpler models of Bjerrum, Ebeling, and Fuoss.

The Bjerrum theory considers only Coulomb interactions between ions. Consider two ions of charge $z_{A}$ and $z_{B}$ such that their centers are the distance *r* apart. The electrostatic energy *U* of these ions, relative to an infinite separation, is given by


$$\begin{array}{c} U=\frac{z_{A}z_{B}e_{0}^{2}}{4\pi \epsilon_{0}\epsilon r}. \end{array}$$ (37)

Bjerrum calculated the differential change in the probability *P* of finding the center of an *A*^+^ ion to be within the range r→r+dr from the center of a *B*^–^ ion as


$$\begin{array}{c} dP=4\pi r^{2}N_{0}[A^{+}]e^{-U/k_{b}T}dr=4\pi r^{2}N_{0}[A^{+}]e^{-z_{A}z_{B}\lambda_{B}/r}dr \end{array}$$ (38)

where the exponential term brings in the Boltzmann distribution. If σ is the distance of closest approach of the ion centers, then the probability of finding an oppositely charged ion between σ and a radial location $r_{B}$ is


$$\begin{array}{c} P_{\sigma \to r_{B}}=4\pi N_{0}[A^{+}]\int_{\sigma }^{r_{B}}r^{2}e^{-z_{A}z_{B}\lambda_{B}/r}dr. \end{array}$$ (39)

The integral in Eq. 39 is divergent for $r_{B}\to \infty$, so a cutoff is conventionally defined as $r_{B}=|z_{A}z_{B}|\lambda_{B}/2$, which is the location where *dP*/*dr* is minimized. With the Bjerrum theory, ions within the range $\sigma \leq r\leq r_{B}$ are considered to be associated while ions with $r>r_{B}$ are not associated. The Bjerrum association constant is then defined as follows


$$\begin{array}{c} K_{B}=4\pi N_{0}\int_{\sigma }^{r_{B}}r^{2}e^{-z_{A}z_{B}\lambda_{B}/r}dr \end{array}$$ (40)

It is noted that $K_{B}\to 0$ if $r_{B}\to \sigma$, which is a result of the integral cutoff.

The theory of Fuoss [41] considered only contact ion pairs. This theory is developed by considering a cation as a charged conducting sphere of diameter σ and an anion as a point charge. An anion is allowed to enter a cation, which forms an ion pair. It is argued that electrostatically, this situation is the same as two spheres in contact with a center-to-center distance σ. The association constant is derived by evaluating the partitioning between associated and free ions as cations and anions are added to the system, resulting in the following expression.


$$\begin{array}{c} K_{F}=\frac{4\pi }{3}N_{0}\sigma^{3}e^{|z_{A}z_{B}|\lambda_{B}/\sigma } \end{array}$$ (41)

This theory avoids the need for a cutoff radius.

The Ebeling theory involves a modification of the Bjerrum treatment to enable the second coefficient in a virial expansion to be recovered. The Ebeling integral is as follows.


$$\begin{array}{c} K_{E}=4\pi N_{0}\int_{\sigma }^{\infty }r^{2}\left[e^{z_{A}z_{B}\lambda_{B}/r}+e^{-z_{A}z_{B}\lambda_{B}/r}-2-{\left(\frac{2\lambda_{B}}{r}\right)}^{2}\right]dr \end{array}$$ (42)

which becomes, upon integration


$$\begin{array}{c} K_{E}=8\pi N_{0}\sigma^{3}J \end{array}$$ (43)

where


$$\begin{array}{c} J=\frac{\theta^{3}}{12}\left[Ei(\theta )-Ei(-\theta )\right]-\frac{1}{3}\cosh(\theta )-\frac{\theta }{6}\sinh(\theta )-\frac{\theta^{2}}{6}\cosh(\theta )+\frac{1}{3}+\frac{\theta^{2}}{2} \end{array}$$ (44)

and


$$\begin{array}{c} \theta =\frac{\lambda_{B}}{\sigma }. \end{array}$$ (45)

The Ebeling association model does not involve use of a cutoff.

**Fig 5:**
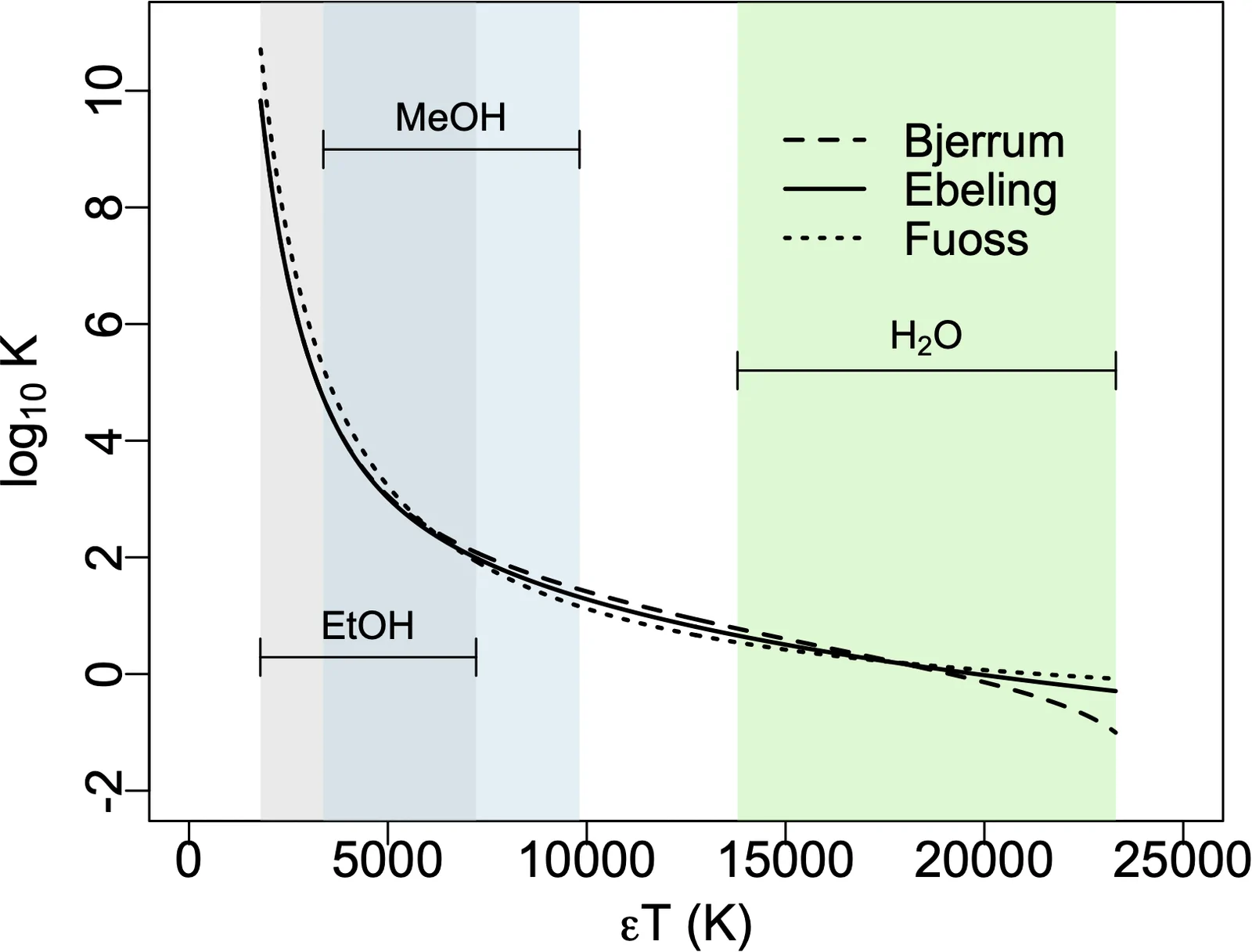
Plots of *K* as a function of ϵT with σ=0.345 nm.

Fig 5 (S5 Fig and S1 File) shows calculated values of *K* as a function of ϵT for these theories with σ=0.345 nm. The values of *K* vary by several orders of magnitude with *K* increasing sharply as ϵT decreases. The theories all agree reasonably well for ϵT less than about 20000 *K*, after which only the Bjerrum and Ebeling theories show reasonable agreement. The applicable ranges for ethanol, methanol, and water are marked as gray, blue, and green shaded bands, respectively, on the figure. There is some overlap between the ethanol and methanol bands while the water band does not overlap with the ethanol and methanol bands.

Table 3 (S1 Table and S1 File) shows values of the association constants for the Bjerrum ($K_{B}$), Ebeling ($K_{E}$), and Fuoss ($K_{F}$) theories with σ=0.345 nm. All of the association constants increase sharply as ϵT decreases. The Bjerrum value is smaller than the other values at ϵT=20000 K. The relative differences are smaller for the other ϵT values in this table, with the Bjerrum and Ebeling theories agreeing closely for ϵT=2000 K.

**Table 3:** Association constants calculated using the Bjerrum, Ebeling, and Fuoss theories for σ=0.345 nm.

ϵT (K)	$K_{B}\;(M^{-1})$	$K_{E}\;(M^{-1})$	$K_{F}\;(M^{-1})$
2000	$5.172\times 10^{8}$	$5.174\times 10^{8}$	$3.448\times 10^{9}$
5000	1130	1045	1675
10000	25.98	19.14	13.17
20000	0.7211	0.9545	1.1678

**Fig 6:**
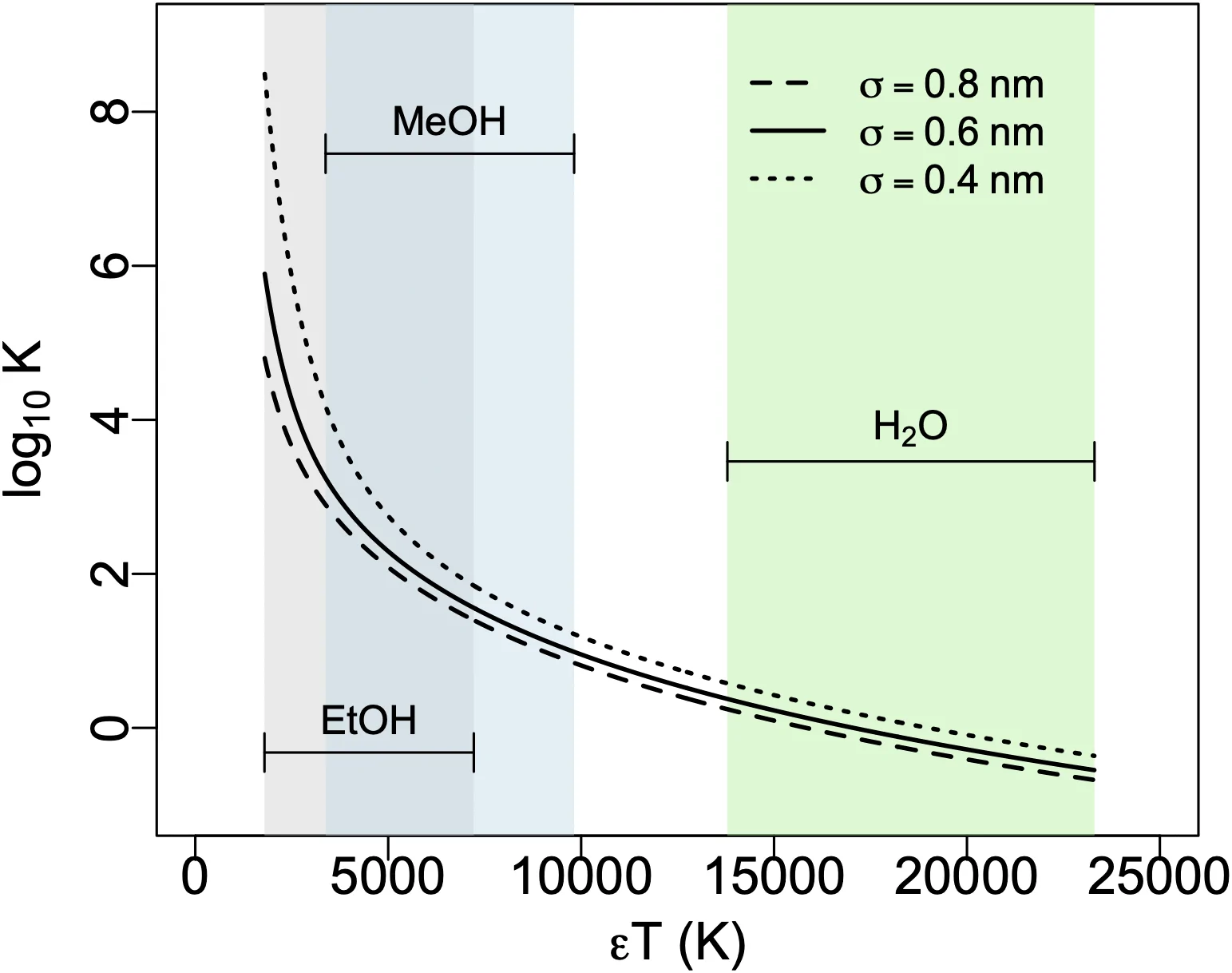
Plots of *K* as a function of ϵT with various σ values for the Ebeling association constant.

Fig 6 (S6 Fig and S1 File) shows plots of the Ebeling association constant as a function of ϵT for different values of σ. As ϵT decreases with fixed σ, the association constant value rises sharply, and for a fixed value of ϵT the association constant can also rise sharply as σ is decreased.

## Results

**Fig 7:**
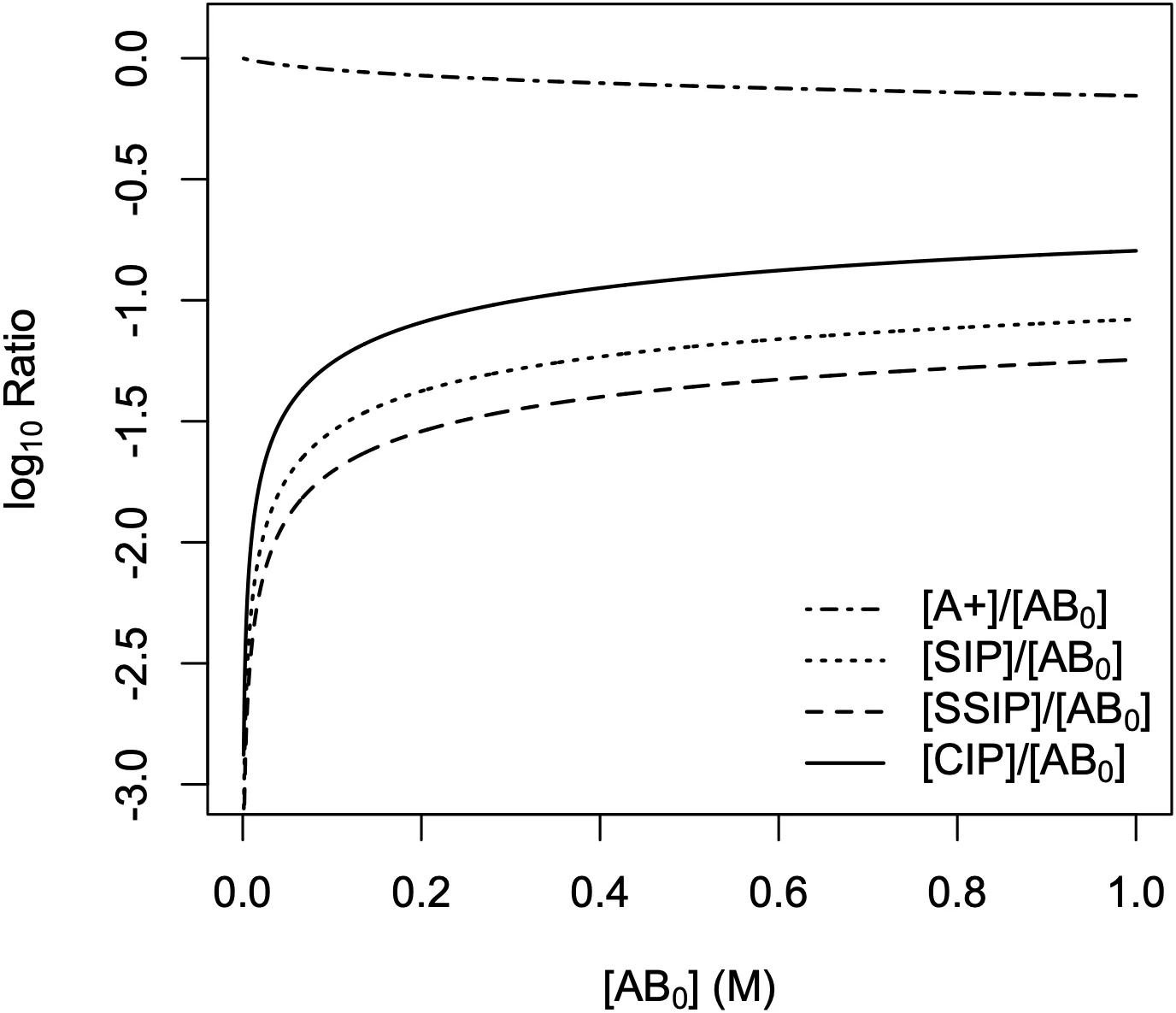
Plots of concentration ratios as a function of [*AB*]_0_ for water with ϵT=18000 K.

Fig 7 (S7 Fig and S1 File) shows plots of the concentration ratios $[A^{+}]/[AB{]}_{0}$, [*SIP*]/[*AB*]_0_, [*SSIP*]/[*AB*]_0_, and [*CIP*]/[*AB*]_0_ as a function of [*AB*]_0_ for the solvent being water and with ϵT=18000 K. The ions $NH_{3}OH^{+}$ and $NO_{3}^{-}$ were assumed to have the same diameter, which is defined to be the arithmetic average of their respective diameters in Table 1. The level of ion pair formation grows rapidly as [*AB*]_0_ is increased from zero until [*AB*]_0_ is close to 0.1 *M*, after which the ion pair concentrations increase slowly with increasing [*AB*]_0_ but never approach unity.

The profiles in Fig 7 were determined by defining the function


$$\begin{array}{c} F([A^{+}],[AB{]}_{0})=f_{\pm }^{2}K[A^{+}{]}^{2}+[A^{+}]-[AB{]}_{0} \end{array}$$ (46)

and then evaluating this function by using 1000 values of [*A*^+^] and 1000 values of [*AB*]_0_ over the ranges $0\leq [A^{+}]\leq 1$ and $0\leq [AB{]}_{0}\leq 1$, corresponding to a resolution of 0.1%. The contour corresponding to $F([A^{+}],[AB{]}_{0})=0$ was then extracted and plotted in the $\left[AB{]}_{0}-[A^{+}\right]$ plane, yielding the solution to Eq. 13. This procedure allowed for the identification of multiple solutions for [*A*^+^] for a given value of [*AB*]_0_. Once the [*A*^+^] values were determined, values for the ion pair concentrations were calculated using Eqs. 9, 10, and 11.

**Fig 8:**
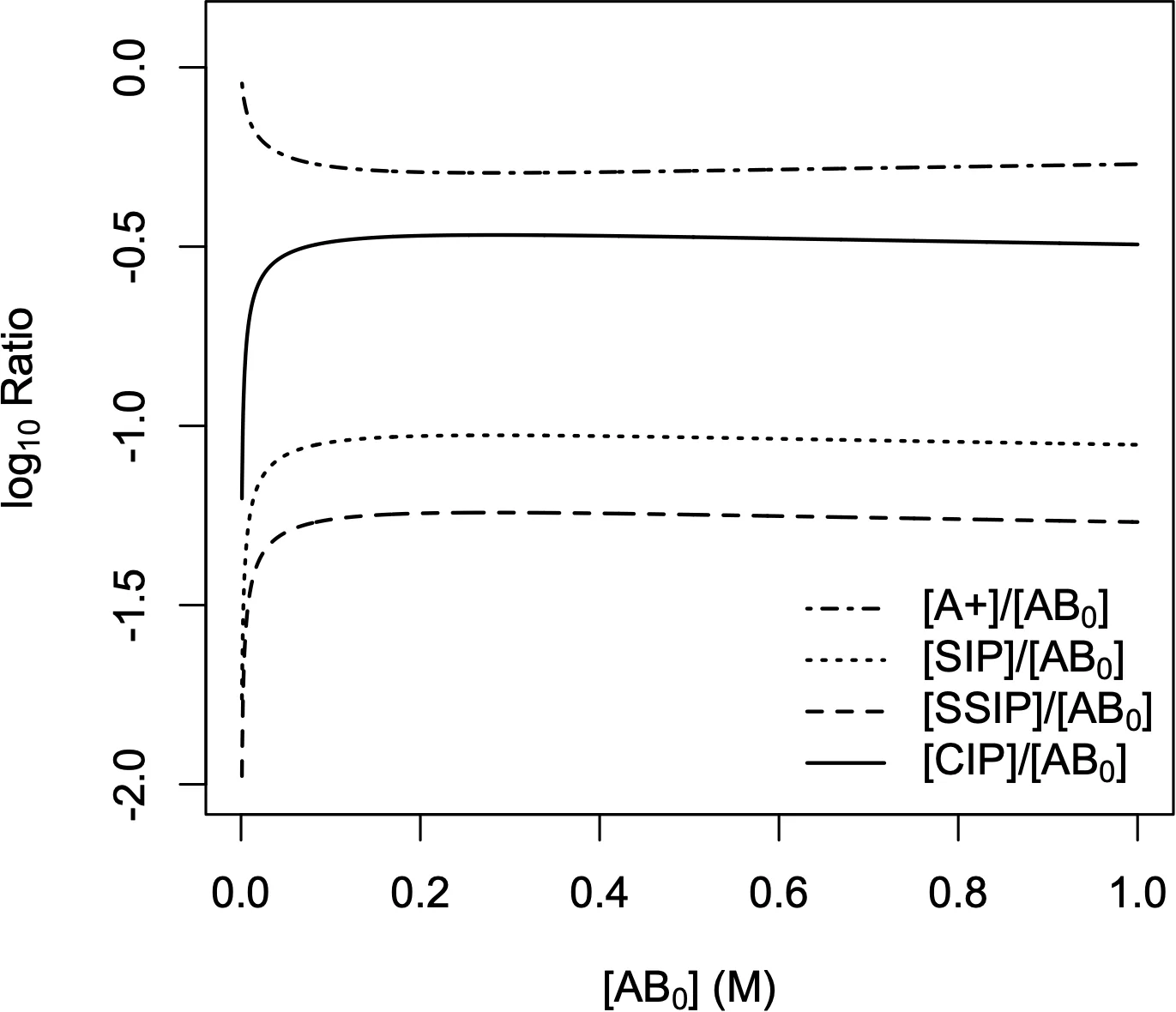
Plots of concentration ratios as a function of [*AB*]_0_ for methanol with ϵT=7000 K.

**Fig 9:**
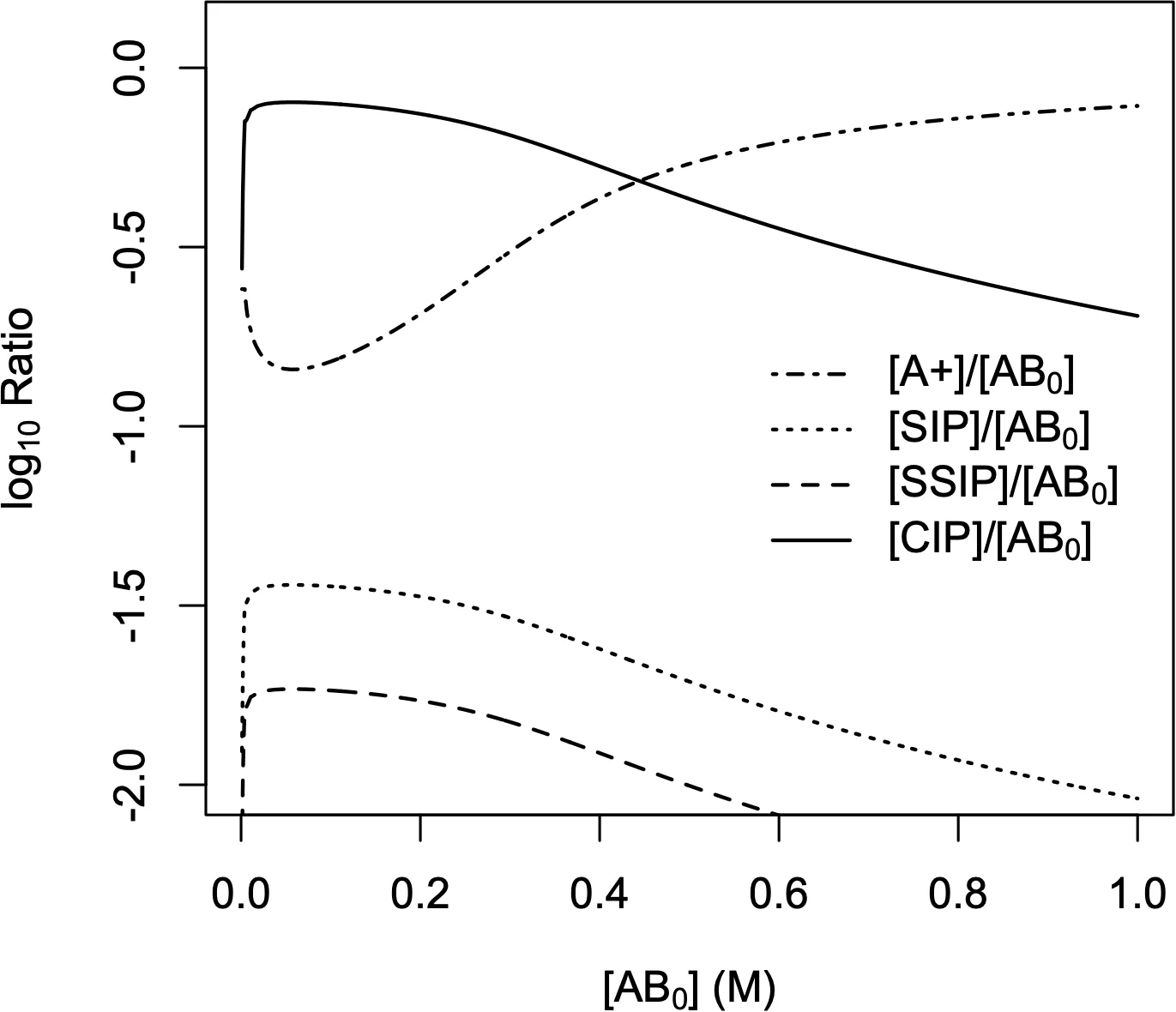
Plots of concentration ratios as a function of [*AB*]_0_ for ethanol with ϵT=4000 K.

Fig 8 (S8 Fig and S1 File) and Fig 9 (S9 Fig and S1 File) show similar data for methanol and ethanol at ϵT=7000 K and 4000 *K*, respectively. These figures show that the fraction of CIP increases rapidly as [*AB*]_0_ is increased from zero. In the case of ϵT=7000 K, a slight downward trend in the ion pair concentration ratios is evident for [*AB*]_0_ greater than about 0.1 *M*. For ϵT=4000 K, the downward trend begins at a smaller [*AB*]_0_ value and the rates of decrease are larger. It is also noted that the SIP and SSIP concentration fractions are small relative to the CIP and [*A*^+^] concentration fractions.

**Fig 10:**
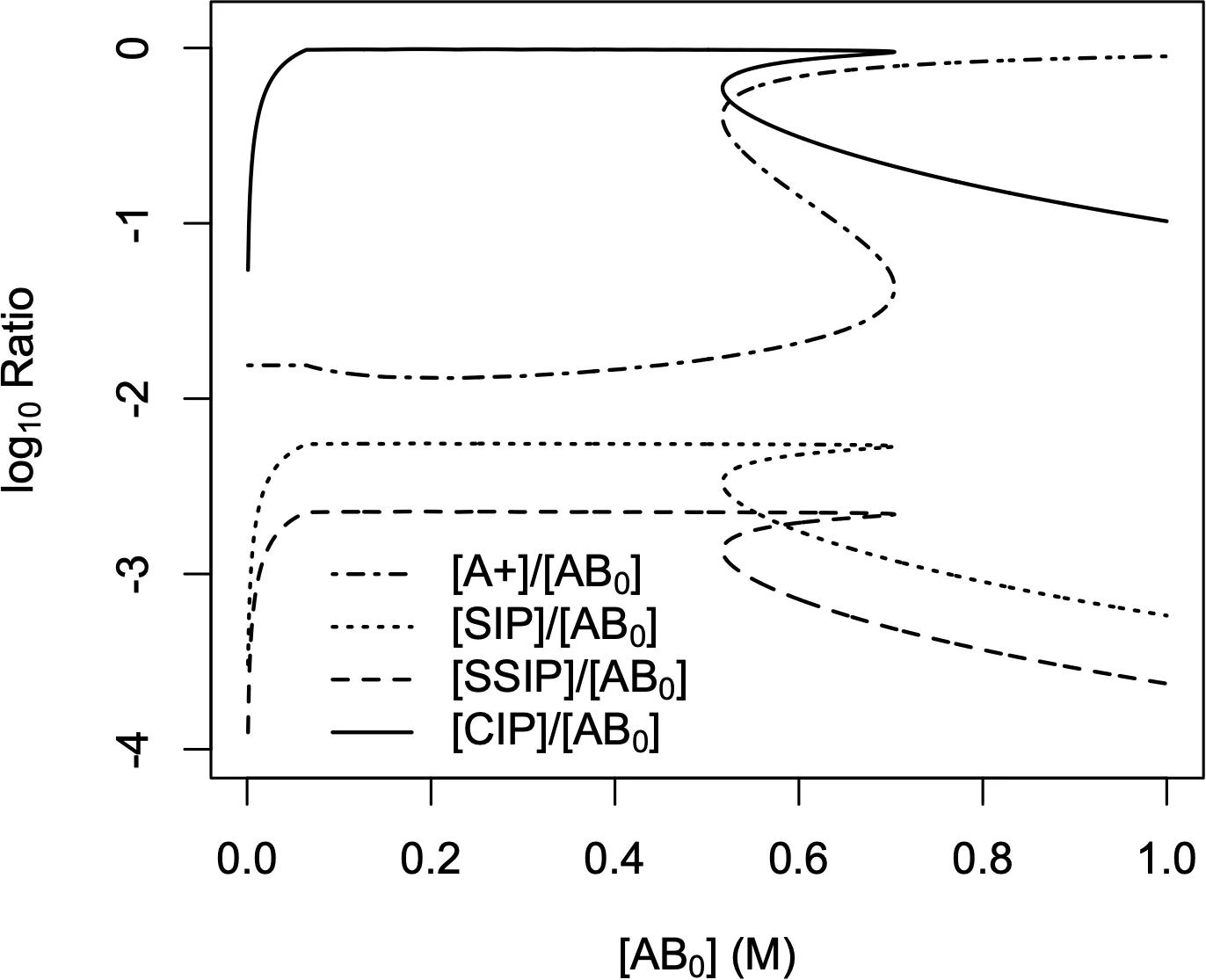
Plots of concentration ratios as a function of [*AB*]_0_ for ethanol with ϵT=3000 K.

Fig 10 (S10 Fig and S1 File) shows plots of the concentration ratios for ethanol with ϵT=3000 K. There is an [*AB*]_0_ range from about 0.55−0.70 M where the profiles are multivalued. This is discussed in more detail below.

Considering that the SIP and SSIP levels are small, we may develop a simplified expression to predict CIPs by neglecting the SIP and SSIP concentrations entirely by setting [*SIP*] = [*SSIP*] = 0. For simplicity of notation, we define *x* = [*AB*]_0_ and *y* = [*CIP*]/[*AB*]_0_. Combining Eqs. 12 and 13 and setting $K=K_{CIP}$ yields the following expression.


$$\begin{array}{c} y=Kx(1-y{)}^{2}f_{\pm }^{2} \end{array}$$ (47)

The relationship between *x* and *y* was evaluated numerically by defining the function


$$\begin{array}{c} G(x,y)=y-Kx(1-y{)}^{2}f_{\pm }^{2} \end{array}$$ (48)

where $f_{\pm }$ is evaluated using I=x(1−y), and then evaluating *G*(*x*,*y*) by using 1000 values of *x* and 1000 values of *y* over the ranges 0≤x≤1 and 0≤y≤1, corresponding to a resolution of 0.1%. The contour corresponding to *G*(*x*,*y*) = 0 was then extracted and plotted in the x−y plane, yielding the solution to Eq. 47. This procedure allowed for the identification of multiple solutions for *y* for a given value of *x*.

**Fig 11:**
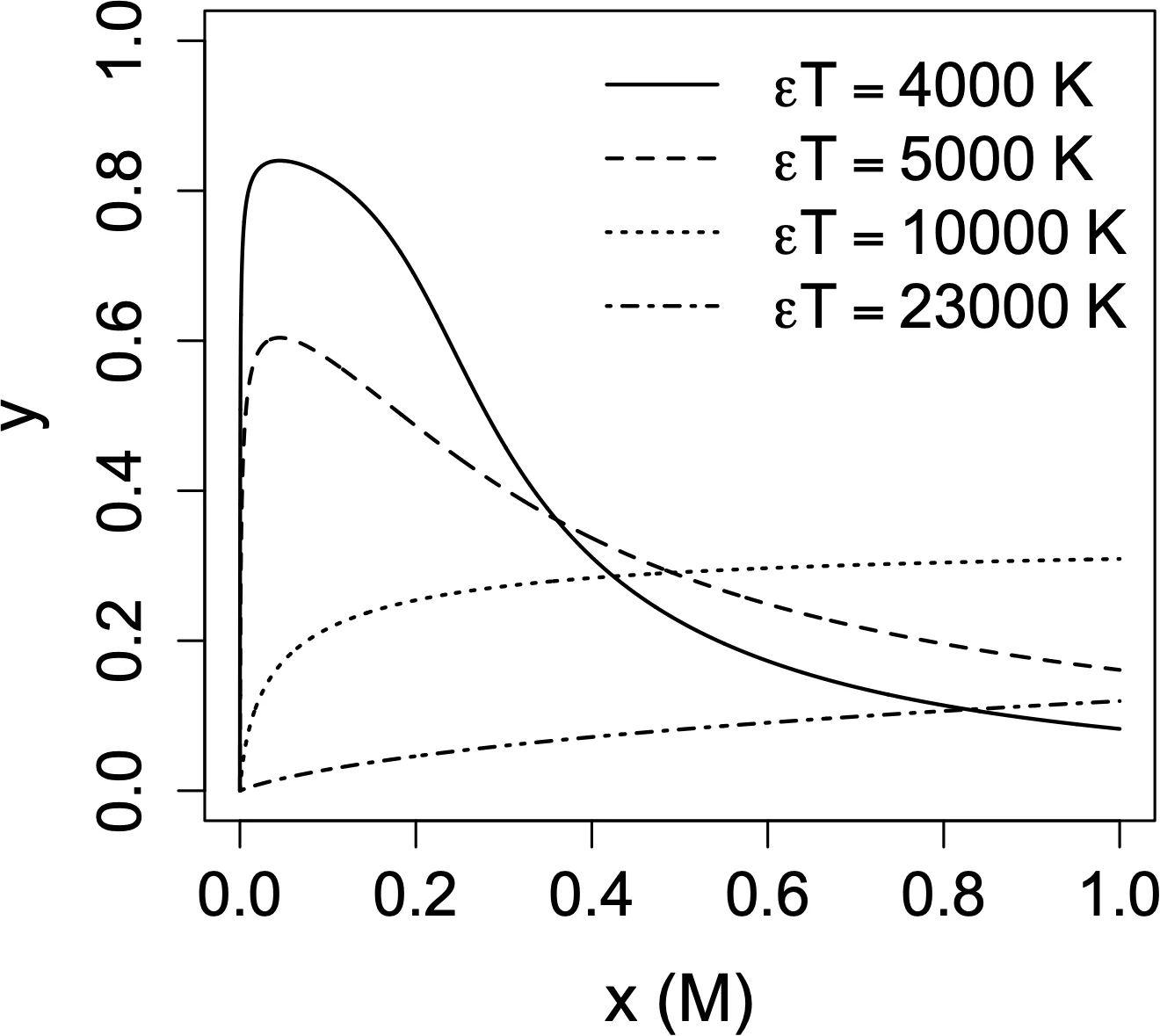
Plots of *y* as a function of *x* for various ϵT values.

Fig 11 (S11 Fig and S1 File) shows plots of *y* as a function of *x* for εT values of 4000 *K*, 5000 *K*, 10000 *K*, and 23000 *K* for the AMSA theory and the Ebeling association constant with σ=0.345 nm. For ϵT=10000 K and 23000 *K*, the *y* values drop monotonically to zero as x→0, where the 23000 *K* values are always below the 10000 *K* values. In contrast, the *y* profiles for ϵT=4000 K and 5000 *K* display peaks at small values of *x*, with the peak being higher and sharper for ϵT=4000 K than for ϵT= 5000 *K*. The data for ϵT=4000 K and 5000 *K* show that a substantial fraction of HAN may form CIPs for small *x* values as long as ϵT is sufficiently small.

**Fig 12:**
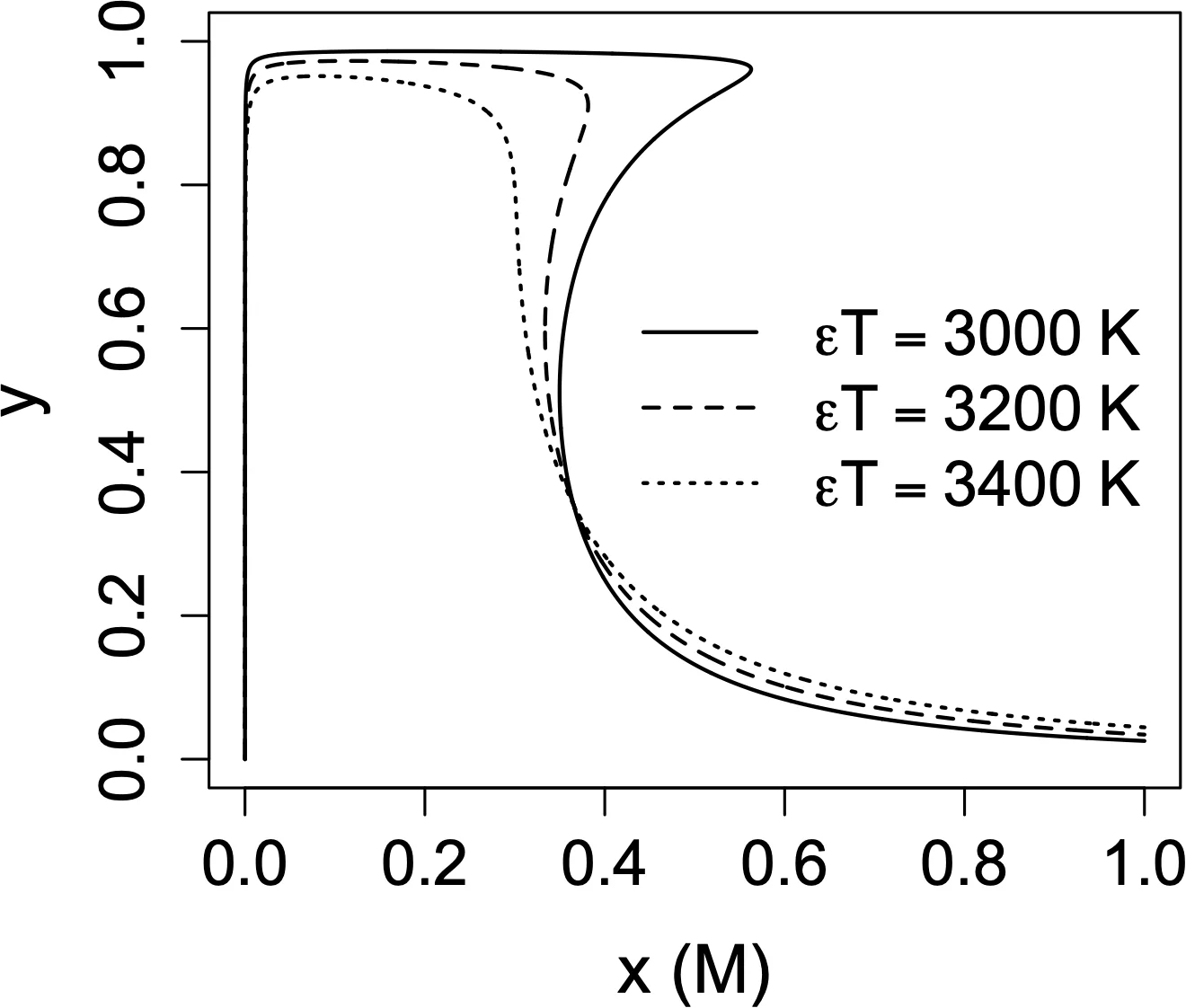
Plots of *y* as a function of *x* for various ϵT values.

Fig 12 (S12 Fig and S1 File) shows plots of *y* as a function of *x* for εT values of 3000 *K*, 3200 *K*, and 3400 *K* for the AMSA theory with the Ebeling association constant (σ=0.345 nm). As ϵT decreases, broad regions appear where *y* is close to unity, i.e., where most of the HAN forms CIPs. In addition, the solution for *y* becomes multivalued, exhibiting multiple branches, if ϵT is sufficiently small.

The shapes of the multivalued curves in Fig 12 are interpreted as follows. We break each curve into four branches, i.e., an initial branch, an upper branch, a middle branch, and a lower branch. Starting from *x* = 0 *M*, the solution for *y* follows the initial branch until *y* closely approaches unity. The initial branch is very steep because *K* is large. Further increases in *x* cause small changes in *y* along the upper branch until a critical value of *x* is attained, after which the solution for *y* would jump to the lower branch, where *y* is small.

If instead we start at *x* = 1 *M* and then decrease *x*, the solution for *y* would smoothly increase until the lower branch ends at about *x* = 0.3 *M*. Further decreases in *x* would cause *y* to jump to the upper branch, after which *y* would follow the upper branch and the initial branch as *x* decreased. If a solution was prepared such that it was initially on the middle branch, then *y* would change monotonically with *x* until either end of the middle branch was reached, after which the solution would jump to another branch. Further research is needed, however, to determine the stability of the branches.

The appearance of multiple solutions for *y* may be understood by defining the variables LHS and RHS as the left-hand and right-hand sides of Eq. 47, respectively, and plotting these variables as a function of *y* for different values of *x* and ϵT. Solutions to Eq. 47 exist at the points where the *LHS* and *RHS* curves intersect.


$$\begin{array}{c} LHS=y \end{array}$$ (49)

$$\begin{array}{c} RHS=Kx(1-y{)}^{2}f_{\pm }^{2} \end{array}$$ (50)

**Fig 13:**
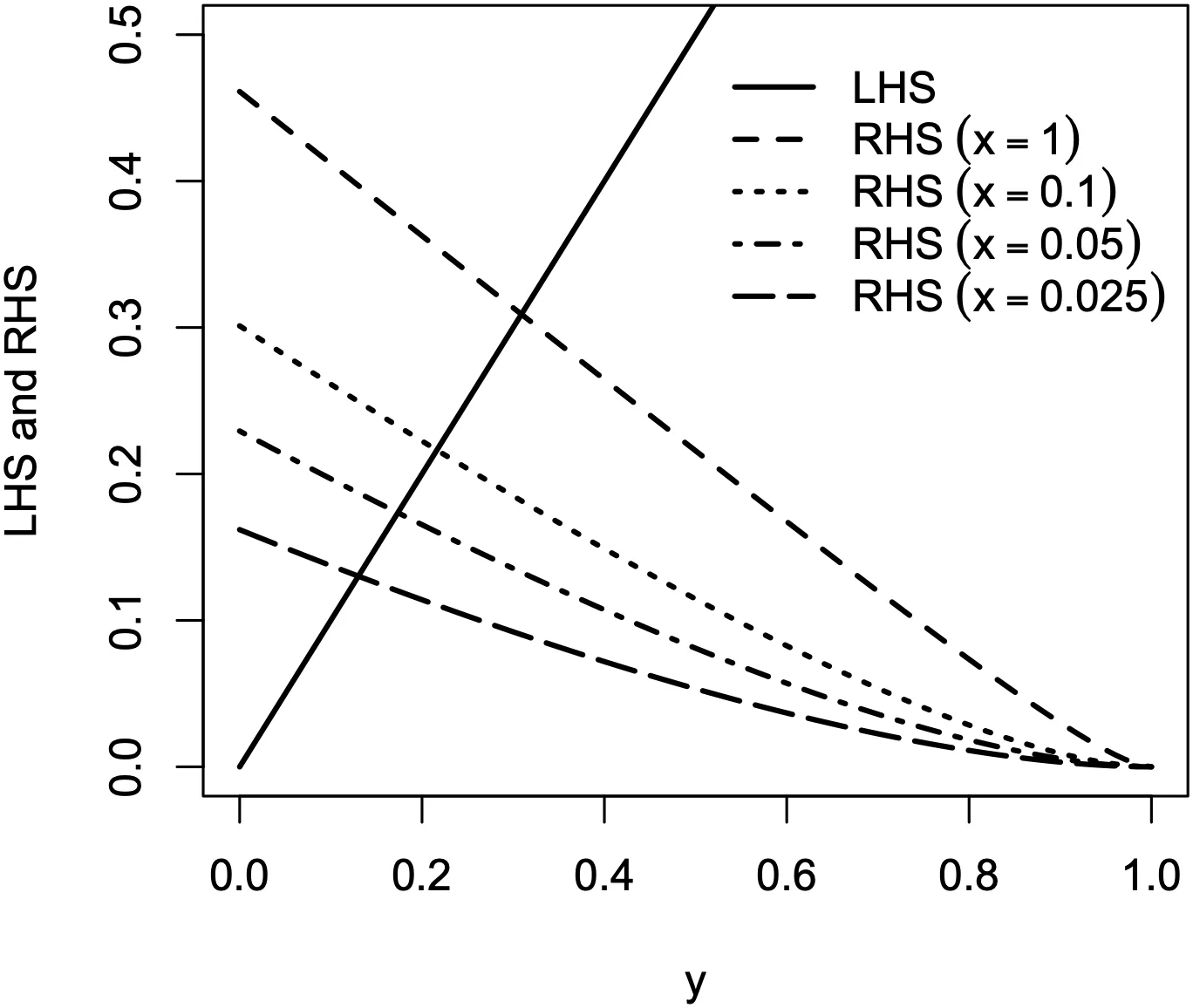
Plots of Eq. 49 and Eq. 50 for ϵT=10000 K.

Fig 13 (S13 Fig and S1 File) shows the LHS and RHS for ϵT=10000 K and various values of *x*. These calculations are based on the AMSA activity coefficient model with the Ebeling association constant (σ=0.345 nm). The LHS intersects each RHS only once, showing that the solutions of Eq. 47 for ϵT=10000 K are unique.

**Fig 14:**
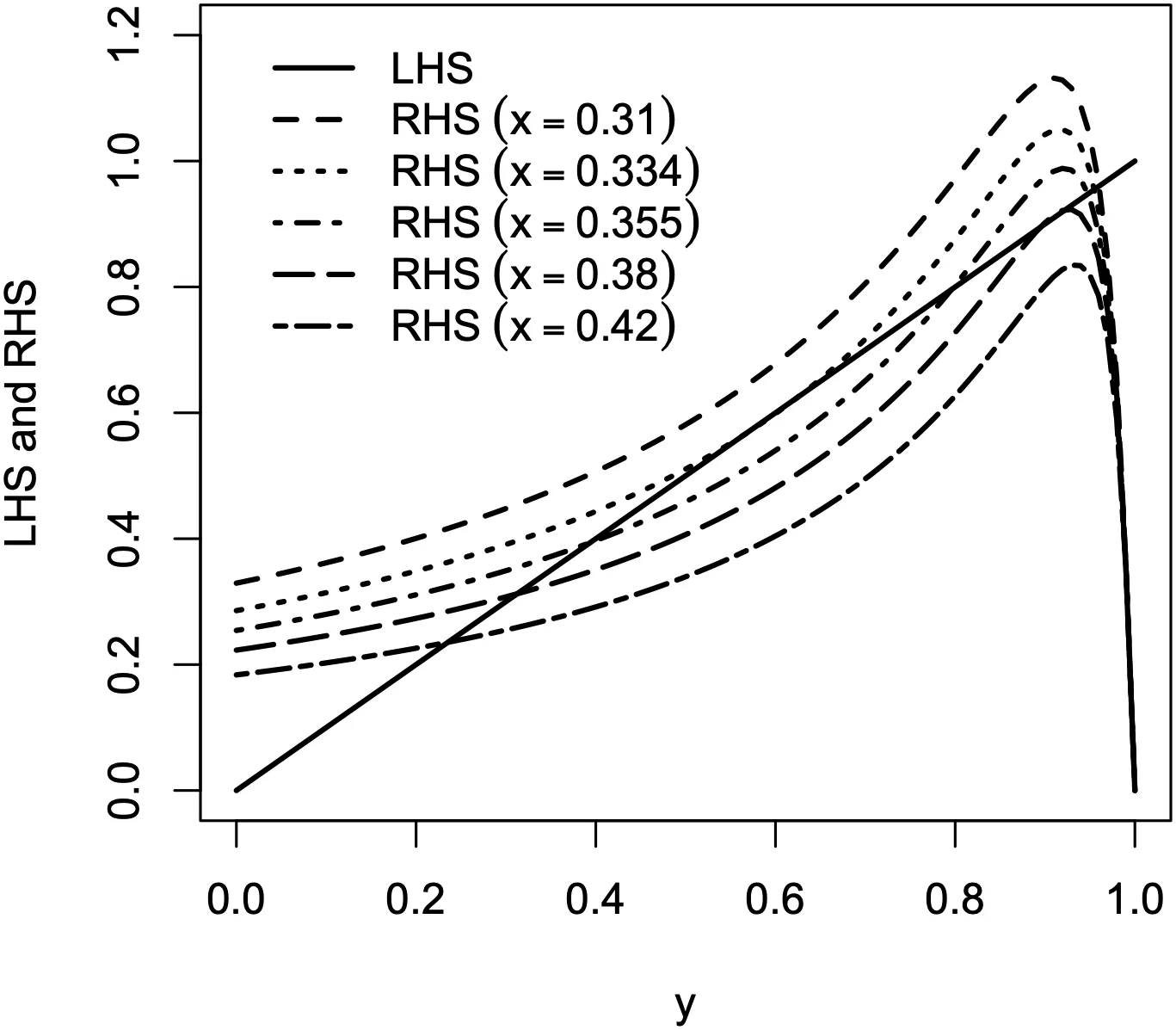
Plots of Eq. 49 and Eq. 50 for ϵT=3200 K.

Fig 14 (S14 Fig and S1 File) shows the LHS and the RHS for ϵT=3200 K and various values of *x*. For *x* = 0.31 *M*, there is only one solution, which is for *y* near unity. When *x* is increased to 0.334 *M*, there are two solutions and this value of *x* may be considered to be a bifurcation point. Further increases in *x* result in three solutions until *x* = 0.38 *M*, where two solutions exist, i.e., a solution near *y* = 1 and a solution near *y* = 0.3. This value of *x* may also be considered to be a bifurcation point. When *x* is larger than 0.38 *M*, there is only one solution, and this solution is not near *y* = 1. For example, if *x* = 0.42 *M* then *y* is near 0.24. Further increases in *x* will lead to decreases in *y*.

**Fig 15:**
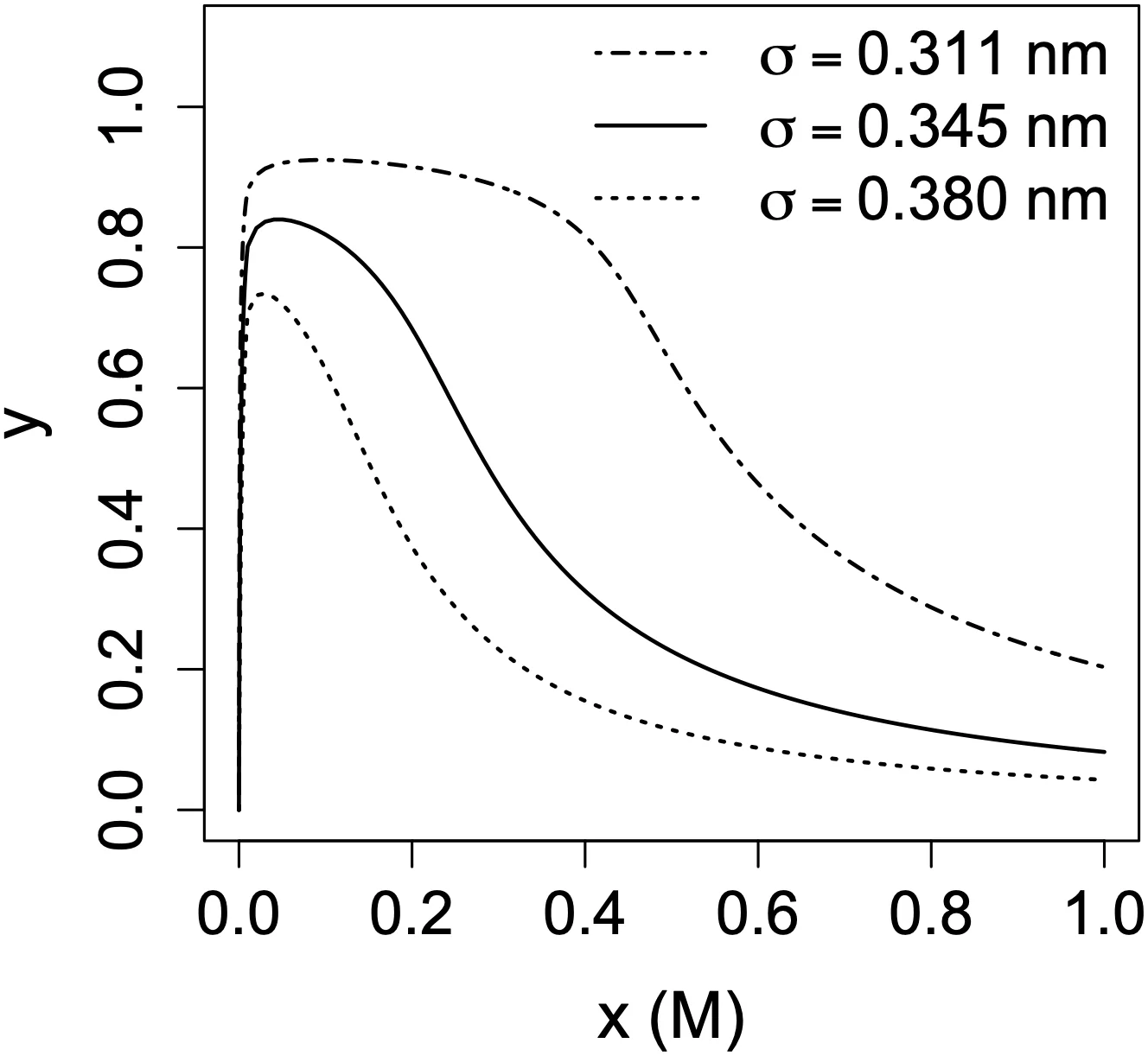
Plots of *y* as a function of *x* for the Ebeling/AMSA model for ϵT=4000 K with different σ values.

The influence of the value of σ was investigated for the AMSA model by varying σ by ±10% around the value 0.345 *nm*. The results are shown in Fig 15 (S15 Fig and S1 File). It was found that *y* values could vary by roughly ±30%, though the location of a peak was not strongly influenced.

**Fig 16:**
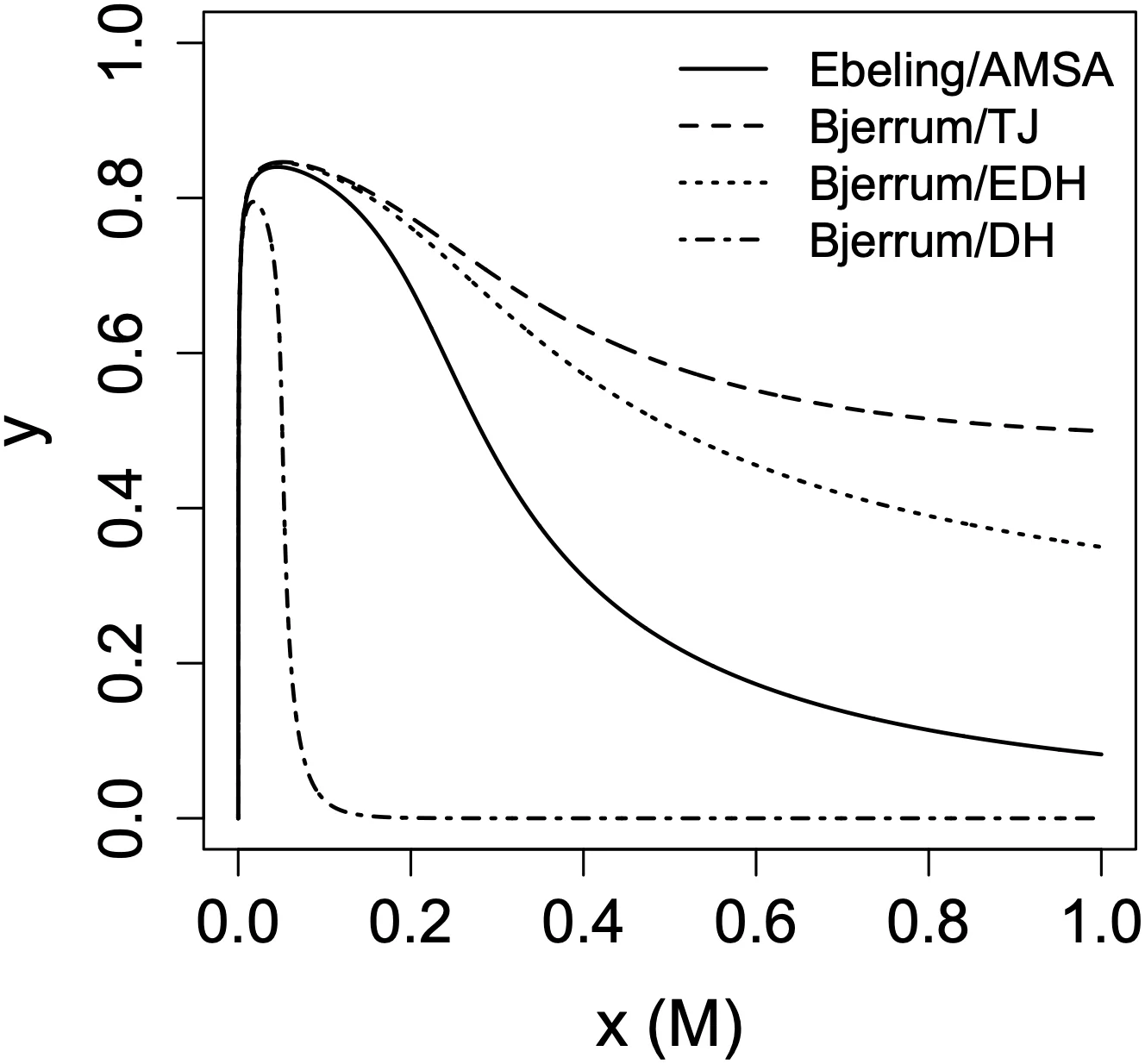
Plots of *y* as a function of *x* for ϵT=4000 K with different association and activity coefficient models.

Fig 16 (S16 Fig and S1 File) shows the effect of using different association and activity coefficient models with ϵT=4000 K. All of the models predict a sharp rise in *y* as *x* is increased from a value of zero, followed by a peak and subsequent decay as *x* is increased further. The peak locations and heights are reasonably close for these different models, but the decay rates after the peaks are quite different.

The trends in Fig 16 are related to the behavior of the activity coefficient models. When *I* << 1, the activity coefficients converge to approximately the same profiles (Fig 4), leading to the closeness of the results prior and up to the peak. After the peak, differences between the activity coefficient models become larger, leading to significant differences in predicted *y* values between the activity coefficient models.

In the limit I→0, all of the activity coefficient models reduce to the Debye-Huckel equation (Eq. 16) in the first approximation. We may then express Eq. 47 for I→0 as follows


$$\begin{array}{c} y\approx Kx(1-y{)}^{2}e^{-2\alpha I^{1/2}}. \end{array}$$ (51)

When $I<<4\alpha^{2}$, the exponential term in Eq. 51 is close to unity, leading to


$$\begin{array}{c} y\approx Kx(1-y{)}^{2}. \end{array}$$ (52)

The solution to Eq. 52 is


$$\begin{array}{c} y\approx 1-\frac{(4Kx+1{)}^{1/2}-1}{2Kx}. \end{array}$$ (53)

Because I=x(1−y), Eq. 53 may be expressed as


$$\begin{array}{c} I\approx \frac{(4Kx+1{)}^{1/2}-1}{2K}. \end{array}$$ (54)

Making use of Eq. 54 then yields


$$\begin{array}{c} 2\alpha I^{1/2}\approx 2\alpha {\left[\frac{(4Kx+1{)}^{1/2}-1}{2K}\right]}^{1/2}. \end{array}$$ (55)

Equations 54 and 55 illustrate why there may be a peak in the *y* profile. As *x* is increased, the ionic strength *I* will also increase until $2\alpha I^{1/2}$ becomes of order unity. Further increases in *x* would lead to further increases in *u*, causing *y* to decrease. Physically, *y* initially increases with *x* because the ionic strength is too low to influence the activity coefficient. As *x* increases, the activity coefficient will eventually become appreciably smaller than unity, leading to the peak.

We now consider conditions where *dy*/*dx* = 0 in more detail. We define $f_{\pm }^{2}=e^{-u}$ such that Eq. 48 becomes


$$\begin{array}{c} G(x,y)=y-Kx(1-y{)}^{2}e^{-u}. \end{array}$$ (56)

Taking the total differential of Eq. 56 yields


$$\begin{array}{c} dG=G_{x}dx+G_{y}dy \end{array}$$ (57)

where $G_{x}$ and $G_{y}$ are the partial derivatives of *G* with respect to *x* and *y*. The differential *dG* is zero along the contour *G* = 0, leading to the following expression for *dy*/*dx*.


$$\begin{array}{c} \frac{dy}{dx}=-\frac{G_{x}}{G_{y}} \end{array}$$ (58)

Differentiating Eq. 48 with respect to *x* and *y* yields


$$\begin{array}{c} G_{x}=-\frac{y}{x}(1-xu_{x}) \end{array}$$ (59)

$$\begin{array}{c} G_{y}=1+\frac{y}{1-y}\left(2+(1-y)u_{y}\right) \end{array}$$ (60)

such that


$$\begin{array}{c} \frac{dy}{dx}=\frac{1}{x}\frac{y(1-y)(1-xu_{x})}{1+y\left(2+(1-y)u_{y}\right)}. \end{array}$$ (61)

An extremum in *y* thus occurs when


$$\begin{array}{c} 1-xu_{x}=0. \end{array}$$ (62)

The extrema must also satisfy


$$\begin{array}{c} y=Kx(1-y{)}^{2}e^{-u}. \end{array}$$ (63)

There are also extrema if x=∞, *y* = 0, or *y* = 1, but these are not of interest here. Equations 62 and 63 allow the values of *x* and *y* corresponding to an extremum, denoted as $x_{c}$ and $y_{c}$, respectively, to be determined, where these values depend on the activity coefficient model.

For the AMSA equation with a common diameter σ and $z_{A}^{2}+z_{B}^{2}=2$ we have


$$\begin{array}{c} u=2\frac{\lambda_{B}}{\sigma }\frac{(1+2\sigma \phi I^{1/2}{)}^{1/2}-1}{(1+2\sigma \phi I^{1/2}{)}^{1/2}+1} \end{array}$$ (64)

where $\kappa =\phi I^{1/2}$ with $\phi =(2e_{0}^{2}N_{0}/\epsilon_{0}k_{b}\epsilon T{)}^{1/2}$. Because I=x(1−y), we may write $xu_{x}=Iu_{I}$, allowing us to utilize Eq. 62 to derive the following expression


$$\begin{array}{c} w^{2}+\left(1-\theta \right)w+\theta =0 \end{array}$$ (65)

where


$$\begin{array}{c} w={\left(1+2\sigma \phi I^{1/2}\right)}^{1/2}. \end{array}$$ (66)

Equation 65 has the following roots.


$$\begin{array}{c} w_{1}=\frac{\theta -1-(\theta^{2}-6\theta +1{)}^{1/2}}{2} \end{array}$$ (67)

$$\begin{array}{c} w_{2}=\frac{\theta -1+(\theta^{2}-6\theta +1{)}^{1/2}}{2} \end{array}$$ (68)

**Fig 17:**
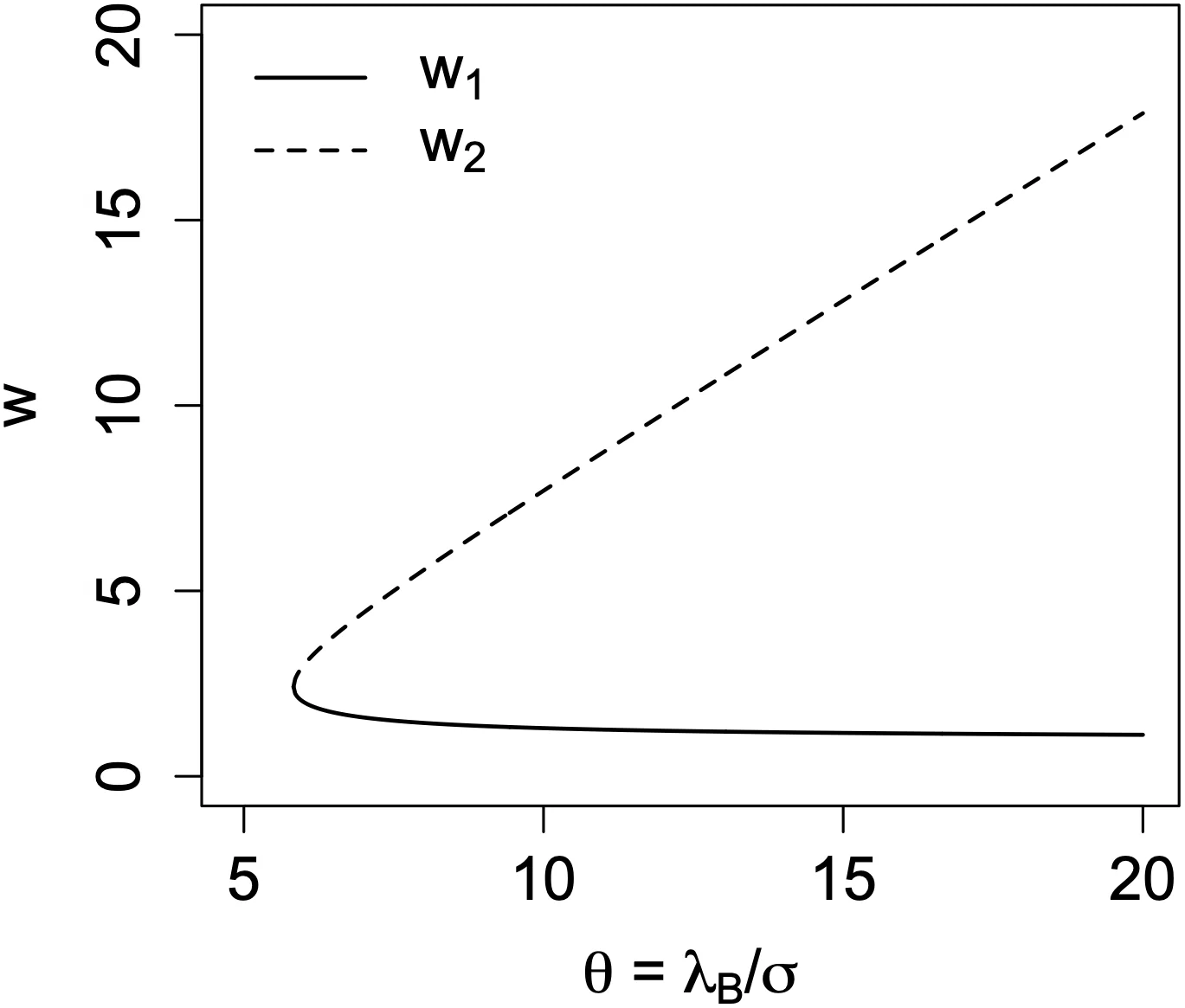
Plot of the roots of Eq. 65 as a function of $\lambda_{B}/\sigma$.

It can be shown that physically relevant values exist for *w*_1_ and *w*_2_ only if $\theta \geq 3+2\sqrt{2}$. If θ is less than this value, the values are complex or real and negative. The roots *w*_1_ and *w*_2_ are plotted in Fig 17 as a function of $\lambda_{B}/\sigma$. The value $\lambda_{B}/\sigma =3+2\sqrt{2}$ corresponds to ϵT=7170 K with σ=0.4 nm. If ϵT is larger than this value, then the AMSA equation predicts that a peak in *y* will not be present. If ϵT is equal to this value, then $w_{1}=w_{2}$ (one root) while if ϵT<7170 K there are two roots as shown in Fig 17 (S17 Fig and S1 File). The smaller root (*w*_1_) corresponds to a maximum while the second root (*w*_2_) corresponds to a minimum. If $w_{1}=w_{2}$, then there is only one extremum, which is a peak. Here we are interested in *w*_1_ because it corresponds to a peak in *y*.

**Fig 18:**
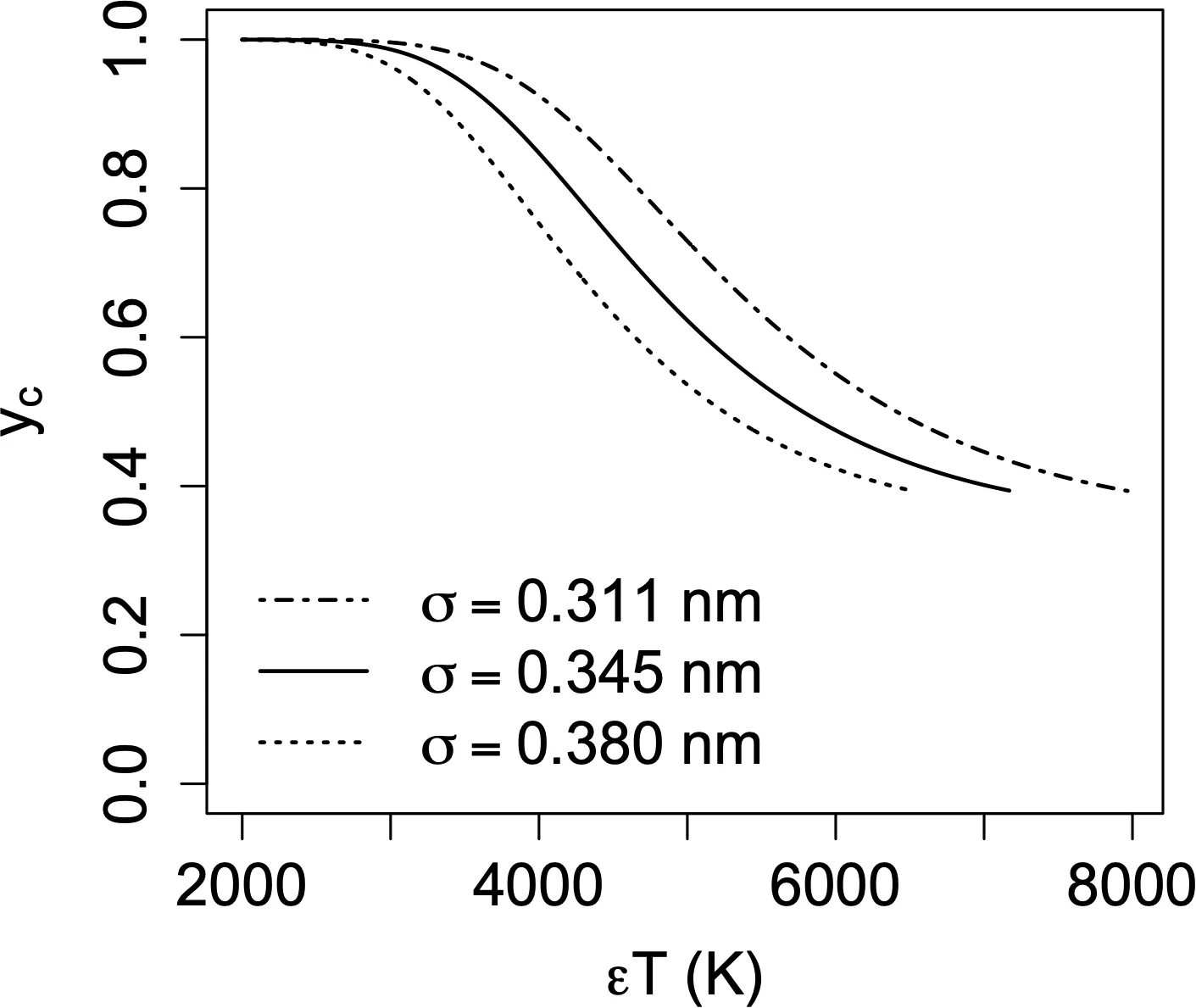
Plots of $y_{c}$ as a function of ϵT with different σ values.

**Fig 19:**
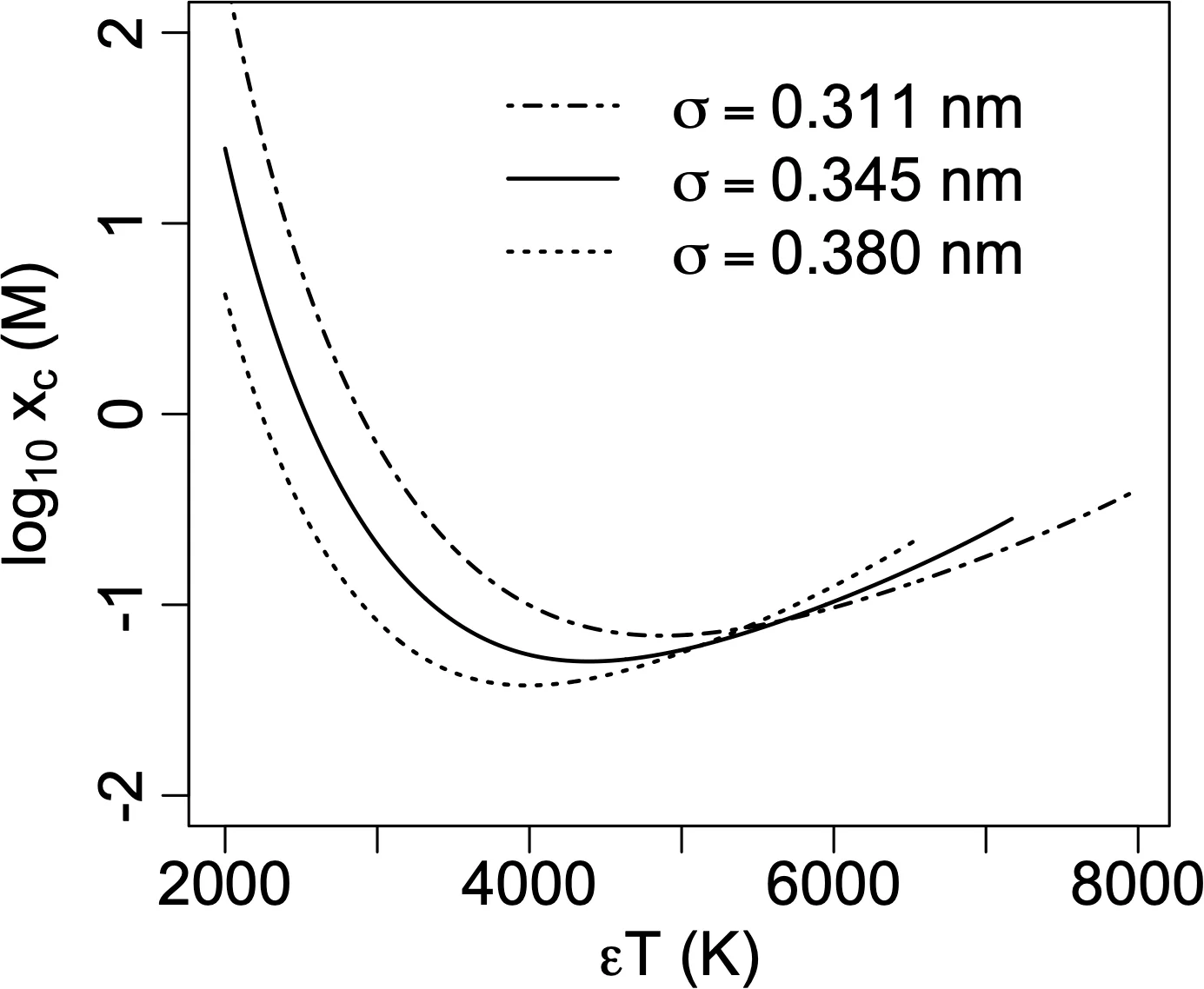
Plots of $x_{c}$ as a function of ϵT with different σ values.

By using the *w*_1_ values from Eq. 67, we may solve for the *x* and *y* values corresponding to peaks, denoted as $x_{c}$ and $y_{c}$, for the AMSA model. The $y_{c}$ results are shown in Fig 18 (S18 Fig and S1 File) where $y_{c}$ is plotted as a function of ϵT for different values of σ. The $y_{c}$ values vary by ≈±0.1 for ϵT near 4000 *K*. Away from this value, the variations in $y_{c}$ become smaller. The corresponding $x_{c}$ results are plotted in Fig 19 (S19 Fig and S1 File) as a function of ϵT.

Similar analyses may be performed for the other activity coefficient models. Like the AMSA model, the EDH and TJ models each predict there is a maximum value of ϵT, beyond which a peak in *y* does not exist. These ϵT values are different from what is predicted by the AMSA model, so the critical ϵT value is dependent on the activity coefficient model. The DH model predicts a peak in *y* will exist for all values of ϵT, i.e., a critical ϵT value does not exist for the DH model.

## Conclusions

This research employed simplified theory to predict CIP concentrations in electrolyte mixtures containing hydroxylammonium nitrate (HAN), considering small HAN loadings in the condensed phase. The theory indicates that CIP concentrations can peak at small, nonzero levels of the initial HAN loading if the product of the liquid dielectric constant and the absolute temperature is sufficiently small. This is relevant to fundamental studies of the chemical kinetics of HAN decomposition, which are often done with dilute mixtures. Performing experiments with low ϵT liquids may result in conditions where CIP concentrations would be appreciable and would have to be accounted for with chemical kinetic mechanisms that are developed for these situations.

It is noted that the present analysis cannot be used for HAN loadings greater than 2−3 M. It is of interest to pursue further research to investigate higher HAN loadings, e.g., using molecular dynamics modeling or extensions of the present theory by employing different activity coefficient models. It is also of interest to relax the continuum dielectric approximation.

Experimental measurements should also be pursued to assess whether CIPs are present as predicted by this theory. For example, electrical conductivity and spectroscopic studies could be used to test whether the predicted CIPs are present at appreciable concentrations in methanol and ethanol. It would also be of interest to perform experiments on HAN decomposition rates in methanol and ethanol to determine whether CIPs have an appreciable influence on HAN decomposition rates.

## Supporting information

S1 Fig Figure_1_Code. (R)

S2 Fig Figure_2_Code. (R)

S3 Fig Figure_3_Code. (R)

S4 Fig Figure_4_Code. (R)

S5 Fig Figure_5_Code. (R)

S6 Fig Figure_6_Code. (R)

S7 Fig Figure_7_Code. (R)

S8 Fig Figure_8_Code. (R)

S9 Fig Figure_9_Code. (R)

S10 Fig Figure_10_Code. (R)

S11 Fig Figure_11_Code. (R)

S12 Fig Figure_12_Code. (R)

S13 Fig Figure_13_Code. (R)

S14 Fig Figure_14_Code. (R)

S15 Fig Figure_15_Code. (R)

S16 Fig Figure_16_Code. (R)

S17 Fig Figure_17_Code. (R)

S18 Fig Figure_18_Code. (R)

S19 Fig Figure_19_Code. (R)

S1 Table Table_3_Code. (R)

S1 File Functions. (R)
